# Genome-Resolved Metagenomics Informs the Functional Ecology of Uncultured Acidobacteria in Redox Oscillated *Sphagnum* Peat

**DOI:** 10.1128/msystems.00055-22

**Published:** 2022-08-29

**Authors:** Linta Reji, Xinning Zhang

**Affiliations:** a Department of Geosciences, Princeton University, Princeton, New Jersey, USA; b High Meadows Environmental Institute, Princeton University, Princeton, New Jersey, USA; Pacific Northwest National Laboratory

**Keywords:** Acidobacteria, metagenomics, peatland biogeochemical cycling, peatland microbiome, soil redox dynamics

## Abstract

Understanding microbial niche differentiation along ecological and geochemical gradients is critical for assessing the mechanisms of ecosystem response to hydrologic variation and other aspects of global change. The lineage-specific biogeochemical roles of the widespread phylum Acidobacteria in hydrologically sensitive ecosystems, such as peatlands, are poorly understood. Here, we demonstrate that Acidobacteria sublineages in *Sphagnum* peat respond differentially to redox fluctuations due to variable oxygen (O_2_) availability, a typical feature of hydrologic variation. Our genome-centric approach disentangles the mechanisms of niche differentiation between the Acidobacteria genera *Holophaga* and *Terracidiphilus* in response to the transient O_2_ exposure of peat in laboratory incubations. Interlineage functional diversification explains the enrichment of the otherwise rare *Holophaga* in anoxic peat after transient O_2_ exposure in comparison to *Terracidiphilus* dominance in continuously anoxic peat. The observed niche differentiation of the two lineages is linked to differences in their carbon degradation potential. *Holophaga* appear to be primarily reliant on carbohydrate oligomers and amino acids, produced during the prior period of O_2_ exposure via the O_2_-stimulated breakdown of peat carbon, rich in complex aromatics and carbohydrate polymers. In contrast, *Terracidiphilus* genomes are enriched in diverse respiratory hydrogenases and carbohydrate active enzymes, enabling the degradation of complex plant polysaccharides into monomers and oligomers for fermentation. We also present the first evidence for the potential contribution of Acidobacteria in peat nitrogen fixation. In addition to canonical molybdenum-based diazotrophy, the Acidobacteria genomes harbor vanadium and iron-only alternative nitrogenases. Together, the results better inform the different functional roles of Acidobacteria in peat biogeochemistry under global change.

**IMPORTANCE** Acidobacteria are among the most widespread and abundant members of the soil bacterial community, yet their ecophysiology remains largely underexplored. In acidic peat systems, Acidobacteria are thought to perform key biogeochemical functions, yet the mechanistic links between the phylogenetic and metabolic diversity within this phylum and peat carbon transformations remain unclear. Here, we employ genomic comparisons of Acidobacteria subgroups enriched in laboratory incubations of peat under variable O_2_ availability to disentangle the lineage-specific functional roles of these microorganisms in peat carbon transformations. Our genome-centric approach reveals that the diversification of Acidobacteria subpopulations across transient O_2_ exposure is linked to differences in their carbon substrate preferences. We also identify a previously unknown functional potential for biological nitrogen fixation in these organisms. This has important implications for carbon, nitrogen, and trace metal cycling in peat systems.

## INTRODUCTION

Acidobacteria constitute an abundant and widely distributed bacterial phylum in terrestrial systems (1–5). They often account for up to 50% of the soil bacterial community (3, 6, 7) across a wide range of habitats, including rhizosphere soils, subsurface aquifer sediments, grassland soils, and wetland environments (4, and references therein). Yet, relatively little is known about the ecophysiological traits that contribute to their success in these environments, largely due to the paucity of cultured representatives.

The physiological characterization of available isolates and community metagenomic investigations suggest vast metabolic diversity within this phylum, ranging from complex carbon transformations to the ability to respire diverse substrates, such as O_2_, sulfate, nitrite, nitrate, and trimethylamine N-oxide (4, 5, 8, 9). Also notable is the extensive phylogenetic diversity within the phylum, with 26 different subdivisions (SD) of Acidobacteria having been defined based on the 16S rRNA gene phylogeny (10), which corresponds to 15 taxonomic classes (11). The majority of the characterized members are within SD 1 (class *Acidobacteriia*, order *Acidobacteriales*) and SD 3 (class *Acidobacteriia*, order *Bryobacterales*) (4, 11, 12). Heterotrophy appears to be a conserved metabolic strategy across all subdivisions. Facultatively and obligately aerobic (including microaerobic) as well as anaerobic members have been described (4, 8, 9, 13–15). Although most of the characterized anaerobic lineages are predominantly fermentative, some are capable of alternative respiration using sulfate or nitrate (5, 8). The isolation conditions for Acidobacteria have generally included the addition of xylan or cellulose as a carbon source, based on the demonstrated ability of many Acidobacteria to utilize these carbohydrates for growth (4, and references therein).

Acidic, sphagnum-dominated peatlands are among the preferred habitats for many Acidobacteria lineages (16–22), and this could be attributed to their vast hydrolytic capabilities, which are associated with the degradation of complex plant-derived polysaccharides (18, 22). Peat organic matter (OM) is predominantly composed of condensed aromatics and plant-derived polymers, such as cellulose, pectin, and hemicellulose, the degradation of which is the primary step toward the anaerobic decomposition of peat OM (23–26). Various hydrolytic extracellular enzymes produced by microorganisms are the key catalytic agents mediating these degradation pathways (24). Their activities, however, may be inhibited by phenolic compounds that are typically abundant in peat systems (27, 28), leading to a mechanistic control on the growth and activity of microbial decomposers, although the evidence for this “enzyme latch” theory is variable (26, 29).

Compared to stable anoxic conditions, the exposure of peat to O_2_ leads to more efficient degradation of complex peat carbon due to the thermodynamic favorability of O_2_ as a highly positive terminal electron acceptor and the requirement of molecular O_2_ in the enzymatic breakdown of phenolic compounds by aromatic compound oxygenases (30–34). Therefore, fluctuating O_2_ levels due to changes in water table position can be a major control on peat carbon stability. In this regard, it is important to consider the effects of projected changes in peat hydrology (28) due to climate change as well as anthropogenic water management strategies on peatland carbon flows. The typically acidic nature of *Sphagnum* peat systems is also considered to be an important control on the complete remineralization of peat OM and methanogenesis (35, 36).

Another major geochemical factor considered to be limiting to microorganisms in organic-rich peat is the low availability of biologically active forms of nitrogen (N), which makes them dependent on atmospheric deposition of N and other biologically important trace metals (e.g., Fe, Cu, Ni, Mo) (37, 38 and references therein), particularly in ombrotrophic systems (39, 40). Diazotrophy has been attributed to *Sphagnum*-associated microorganisms as well as to free-living Cyanobacteria, methanotrophs, and heterotrophic bacteria across peat systems (38, 39, 41–47), although the relative contribution from each functional guild remains largely unknown. Despite their dominance in peat systems, Acidobacteria have not been implicated in biological nitrogen fixation (BNF), and experimental validation for N fixation by the isolate *Holophaga feotida*, which possesses genes for molybdenum-based diazotrophy, is lacking (4).

Recent studies implicate Acidobacteria as key degraders of complex OM in *Sphagnum* peatlands, as many are capable of utilizing glucuronic and galacturonic acids released during *Sphagnum* moss decomposition (17). Acetate is the typical end product of these fermentative reactions, which are mediated by various glycoside hydrolases (20, 21, 48). Metagenomic analyses suggest that many Acidobacteria may further couple acetate oxidation with the reduction of O_2_ or humic substances and may thereby play a key role in the complete mineralization of *Sphagnum*-derived carbon to carbon dioxide (CO_2_) (22). Whether the carbon flow from complex OM to CO_2_ is mediated by all Acidobacteria or by distinct subgroups within the phylum remains unclear, as we lack a lineage-resolved characterization of these organisms. Ecological niche differentiation has previously been observed among Acidobacteria in tundra soil, as the classes *Blastocatellia* and *Acidobacteriia* were found to prefer distinct niche spaces defined by varying pH and carbon substrates (12). Any such niche partitioning along phylogenetic boundaries among peat Acidobacteria, which would result in ecological and metabolic differentiation between lineages, remains largely unknown.

The majority of the phylogenetic diversity within peat Acidobacteria is partitioned among three class level lineages: *Acidobacteriia* (primarily SD 1), *Blastocatellia* (SD 4), and *Holophagae* (SD 8). Within *Acidobacteriia*, the SD 2 and SD 3 lineages are also detected in acidic peat bogs, albeit less frequently (17, 49, and references therein). Even though several cultivated representatives exist within these subdivisions, particularly within SD 1 and SD 4 (e.g., *Granulicella* sp. in SD 1 and *Blastocatella famidurans* A22 in SD 4; see [4] for a recent compilation of cultivated isolates), no representative isolates of peat Acidobacteria exist. This limits inference on their ecophysiological adaptations.

One way to probe the lineage-resolved ecophysiology of uncultivated microbes is to assess if an environmental change elicits differential responses among subpopulations. Given the documented diversity in O_2_ preferences among even closely related populations of Acidobacteria (4), we hypothesized that changes in peat redox will lead to differential enrichment of sublineages. Indeed, when anoxic peat soils were subjected to a temporary increase in O_2_ levels, an increasing dominance of Acidobacteria, specifically the genus *Holophaga*, was observed across incubation time points after anoxia was reestablished (26). The significant restructuring of the microbial community in response to O_2_ exposition ultimately led to a large (~2000-fold) increase in the methane yields of O_2_-shifted samples compared to continuously anoxic controls (26). Geochemical and molecular evidence pointed to O_2_ facilitating the breakdown of complex aromatic lignin and tannin-like carbon compounds, followed by hydrogen (H_2_)-evolving and CO_2_-evolving fermentation, and eventually leading to the proliferation of the genus *Methanobacterium*, which utilized this higher substrate flux (i.e., H_2_ and CO_2_) to generate methane (26). While Acidobacteria, particularly *Holophaga* that were enriched in the O_2_-pretreated incubations, appeared to be key members of the microbial community following oxygenation, their specific roles in the biogeochemical response to transient oxygenation remained unclear.

Here, we apply genome-centric metagenomic approaches to better constrain the functional roles of individual microbial lineages/populations in biogeochemical transformations driven by transient O_2_ exposure in peat incubations. By examining the genomic features of populations enriched across various stages of the incubation (26), we obtain an improved mechanistic understanding of the community succession patterns underlying redox-associated biogeochemical responses, particularly the functional roles of specific Acidobacteria lineages in eliciting these responses.

## RESULTS

### Differential enrichment of uncultured Acidobacteria lineages in oxygen-oscillated peat.

To better understand the functional ecology of peat Acidobacteria, we performed genome assemblies of metagenomic data sets (*n* = 20) obtained from laboratory incubations of *Sphagnum* peat subjected to variable redox oscillations that were associated with shifts in O_2_ levels (26). These peat incubations consisted of triplicate slurry microcosms representing the unsaturated (UNS), above water table (AWT), and below water table (BWT) peat layers. The control slurries were incubated under anoxic conditions throughout the course of the experiment (232 days), while the treatment incubations were pretreated with 5 or 10% O_2_ in the bottle headspace (vol/vol; 98 days), followed by continuously anoxic conditions (134 days).

Pronounced changes in the overall community composition were observed between the different peat layers at the final time point (Fig. S1). Hence, metagenome data sets corresponding to each peat layer at T5 (day 232) were coassembled to improve binning accuracy. Acidobacteria comprised more than 50% of the overall microbial community in each metagenome, and their relative abundance increased in each peat layer, post O_2_ exposure (Fig. S1; Wilcoxon signed-rank test with a *P*-value of <0.05). In total, 12 medium-quality to high-quality (i.e., >70% complete with <10% redundancy) metagenome-assembled genomes (MAGs) of Acidobacteria were obtained (Table 1). Five of these were classified as the genus *Terracidiphillus*, and the remaining seven were classified as *Holophaga*. These two genera fall within SDs 1 and 8, classes *Acidobaceriia* and *Holophagae*, respectively. The *Terracidiphillus* MAGs were obtained from the UNS and BWT peat layers, predominantly from the continuously anoxic incubations. One of the five *Terracidiphilus* genomes (i.e., 10BWT4) was obtained from a redox-oscillated (i.e., 10% O_2_ pretreatment) BWT layer metagenome. In contrast, all seven *Holophaga* MAGs were obtained from O_2_-pretreated incubations of the three peat layers. We adopt the following naming convention for the MAGs: “oxygen pretreatment-peat layer-MAG ID”; for example, ‘0BWT1’ refers to MAG number 1 obtained from a BWT metagenome coassembly from the continuously anoxic (0% O_2_ pretreatment) incubations.

**TABLE 1:** Metagenome-assembled genome statistics and taxonomic classifications^*a*^

MAG	Contigs	Completion %	Redundancy %	Domain	Phylum	Class	Order	Family	Genus	Species
0BWT1	42	99.56	0	Bacteria	Acidobacteriota	Acidobacteriae	Acidobacteriales	Acidobacteriaceae	*Terracidiphilus*	ND
0BWT2	747	86.17	5.36	Bacteria	Acidobacteriota	Acidobacteriae	Acidobacteriales	Acidobacteriaceae	*Terracidiphilus*	ND
0UNS4	36	97.84	0.862	Bacteria	Acidobacteriota	Acidobacteriae	Acidobacteriales	Acidobacteriaceae	*Terracidiphilus*	ND
0UNS5	274	93.53	1.873	Bacteria	Acidobacteriota	Acidobacteriae	Acidobacteriales	Acidobacteriaceae	*Terracidiphilus*	ND
10AWT2	551	94	6.213	Bacteria	Acidobacteriota	Holophagae	Holophagales	Holophagaceae	*Holophaga*	ND
10BWT3	936	83.5	1.754	Bacteria	Acidobacteriota	Holophagae	Holophagales	Holophagaceae	*Holophaga*	ND
10BWT4	718	75.29	3.663	Bacteria	Acidobacteriota	Acidobacteriae	Acidobacteriales	Acidobacteriaceae	*Terracidiphilus*	ND
10UNS1	1067	80.7	0	Bacteria	Acidobacteriota	Holophagae	Holophagales	Holophagaceae	*Holophaga*	ND
5AWT2	143	95.33	0.877	Bacteria	Acidobacteriota	Holophagae	Holophagales	Holophagaceae	*Holophaga*	ND
5AWT5	348	79.4	2.85	Bacteria	Acidobacteriota	Holophagae	Holophagales	Holophagaceae	*Holophaga*	ND
5BWT7	948	92.21	4.191	Bacteria	Acidobacteriota	Holophagae	Holophagales	Holophagaceae	*Holophaga*	ND
5UNS4	305	96	1.151	Bacteria	Acidobacteriota	Holophagae	Holophagales	Holophagaceae	*Holophaga*	ND

a ND, not determined.

10.1128/msystems.00055-22.3 FIG S1 Phylum-level relative abundances of bacteria and archaea in the peat incubation metagenomes, inferred based on *rpoB* gene relative abundances. Abbreviations refer to peat layers and are as follows: UNS, unsaturated; AWT, above water table; and BWT, below water table. The metagenomes from the final time point are highlighted in red boxes. Download FIG S1, TIF file, 2.7 MB. Copyright © 2022 Reji and Zhang. 2022 Reji and Zhang. https://creativecommons.org/licenses/by/4.0/ This content is distributed under the terms of the Creative Commons Attribution 4.0 International license.

**FIG 1:**
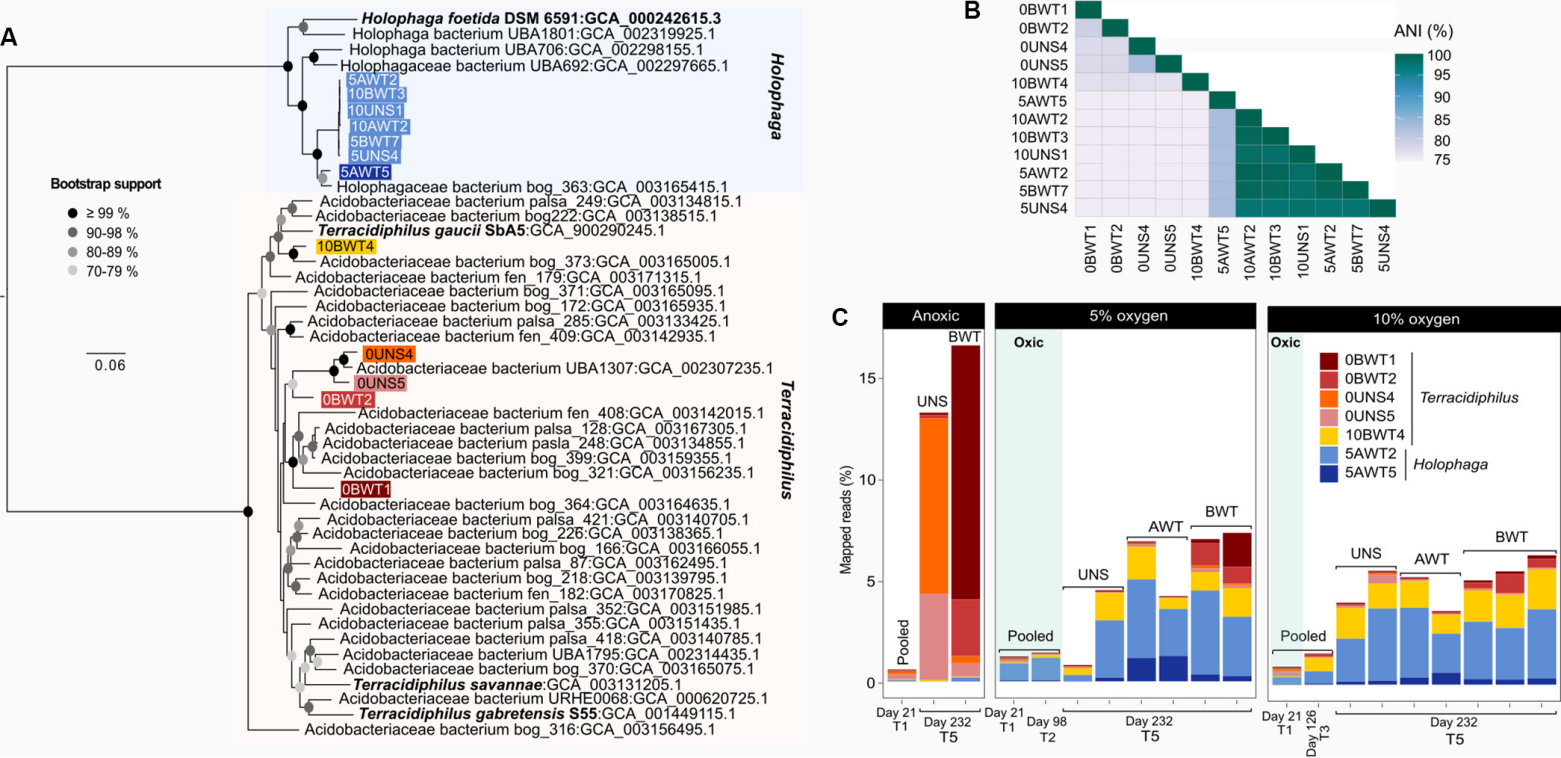
(A) Maximum likelihood phylogenomic tree inferred using a concatenated alignment of select ribosomal proteins. See Materials and Methods for details. Bootstrap support values are indicated at each node using shaded circles. Bold text represents genomes of characterized isolates. Black, unhighlighted leaves represent publicly available Acidobacteria MAGs downloaded from GenBank. (B) Average nucleotide identity (ANI) comparison among the MAGs assembled here. (C) The relative abundance of each lineage was inferred using metagenome read recruitment against the MAGs. For the *Holophaga* species cluster, which shares ~99% ANI, a single MAG 5AWT5 was chosen for read recruitment. Blue shading indicates the oxic part of the experiment in which treatment incubations were purged with O_2_. Due to the small amount of material recovered for molecular analysis at the time points other than T5 (i.e., day 232), samples from each peat layer were pooled for sequencing (indicated as “Pooled”). UNS, unsaturated; AWT, above water table; and BWT, below water table. See (26) for detailed descriptions of the layers.

On a concatenated ribosomal protein phylogenomic tree, the SD 8 *Holophaga* MAGs formed a monophyletic sister clade to the previously described *Holophaga*, which suggests that the MAGs assembled here represent novel, uncharacterized *Holophaga* lineages (Fig. 1A). Six of these MAGs represent a species-level cluster, sharing >97% average nucleotide identity (ANI) among them (Fig. 1B). The seventh MAG, 5AWT5, shares up to 82% ANI with the remaining *Holophaga* genomes (Fig. 1B), and, therefore, likely represents a closely related species. To avoid redundancy in functional comparisons, we limit later analyses to three MAGs within the *Holophaga* cluster, namely, 5AWT5, 5AWT2, and 5UNS4, chosen based on genome quality estimates (Table 1) and phylogenetic novelty (i.e., likely representing distinct, uncharacterized lineages) (Fig. 1A).

The SD 1 *Terracidiphilus* MAGs were more phylogenetically diverse than the *Holophaga* MAGs (Fig. 1A) and shared a relatively lower ANI of 78% with each other (Fig. 1B). Similar to the *Holophaga* MAGs, the *Terracidiphilus* MAGs represent uncultivated lineages, as indicated by their relative phylogenetic divergence from cultivated members within SD 1 (i.e., *Terracidiphilus gaucii* SbA5, *Terracidiphilus savannae*, and Terracidiphilus gabretensis) (Fig. 1A). The majority of the reference genomes within the *Terracidiphilus* genus cluster are MAGs assembled from permafrost metagenomes from the Stordalen Mire in Sweden (21). Three MAGs assembled in our study, 0BWT2, 0UNS4, and 0UNS5, represent particularly undersampled lineages within *Terracidiphilus* as a single reference genome, UBA1307 (assembled from a wetland surface sediment metagenome in [50]; JGI project ID: 1079308), clustered with these MAGs in the phylogenomic tree (Fig. 1A).

In order to understand finer-scale variations in the abundances of the Acidobacteria sublineages, we recruited metagenomic reads against each MAG and compared the percentages of mapped reads across sampling time points as crude proxies for their relative abundances (Fig. 1C). In the continuously anoxic incubations, *Terracidiphilus* MAGs recruited 13 to 16% of the total metagenomic reads at the final time point, representing an approximately 900% increase in relative abundance from the beginning of the incubation, when these MAGs together recruited only ~1.5% of the total reads across all peat layers (Fig. 1C). In stark contrast to the anoxic control incubations, the peat samples pretreated with O_2_ yielded a different taxonomic profile, as these latter incubations were progressively enriched in *Holophaga*. Accordingly, the *Terracidiphilus* MAGs recruited a considerably lower proportion of reads from the redox-oscillated metagenomes compared to the anoxic controls (Fig. 1C).

While a redox-related differential enrichment of the *Terracidiphilus* and *Holophaga* was evident in each peat layer tested, there were notable differences in the population structures of the two groups between peat layers. The continuously anoxic UNS and BWT layers appeared to harbor phylogenetically distinct *Terracidiphilus* populations at the end of the experiment (Fig. 1C). In the unsaturated layer, over 12% of the total mapped reads recruited against the MAGs 0UNS4 and 0UNS5 at the final time point (Fig. 1C). In contrast, the BWT layer appeared to be dominated by the lineages 0BWT1 and 0BWT2 (Fig. 1C). Moreover, a different *Terracidiphilus* lineage, represented by the MAG 10BWT4 (Fig. 1A), appeared to dominate in the O_2_-pretreated samples (Fig. 1C). This genome recruited 2 to 3% of the total reads in each layer in the O_2_-shifted incubations while only recruiting <1% of the reads in the anoxic control incubations. The O_2_-pretreated BWT layers still harbored the *Terracidiphilus* lineages found to be abundant in the anoxic BWT metagenomes (i.e., 0BWT1 and 0BWT2), albeit at notably lower abundances (Fig. 1C). Interlayer differences were much less pronounced for *Holophaga*, even though the relatively divergent 5AWT5 appeared to be more abundant in the AWT layer than in the UNS and BWT layers, particularly in the incubations that were pretreated with 5% O_2_ in the headspace (Fig. 1C).

### Metabolic differentiation among Acidobacteria subpopulations.

**FIG 2:**
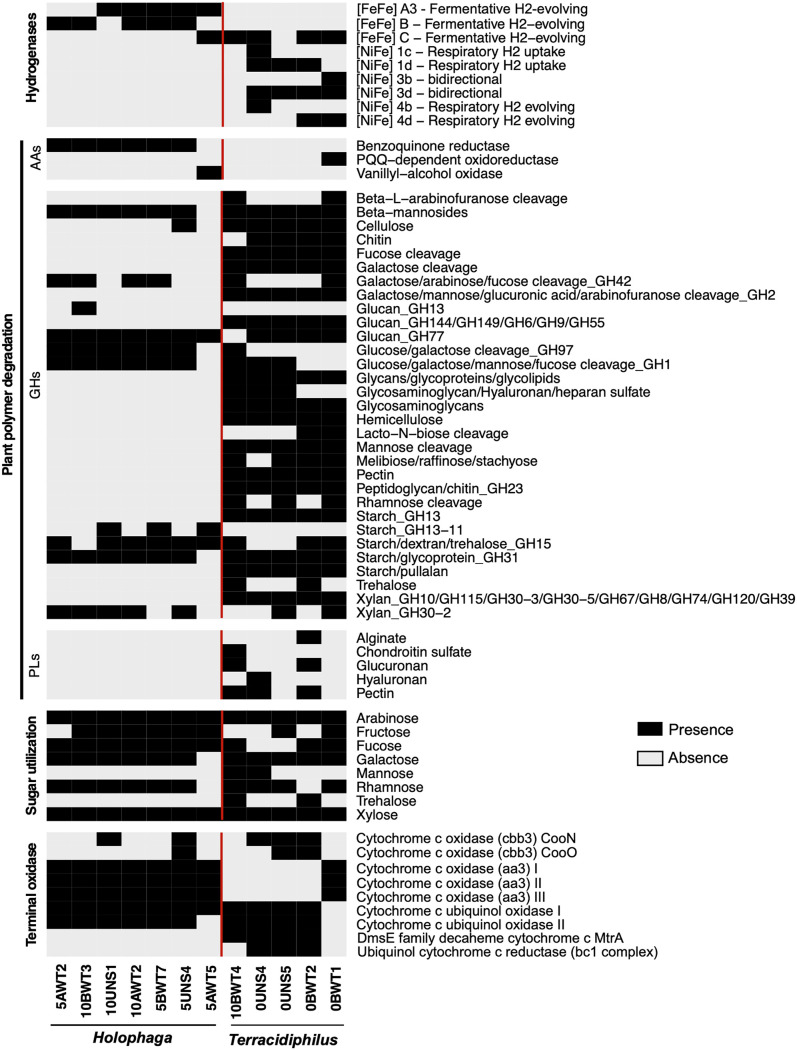
Selected metabolic features across the assembled genomes. CAZyme profiles are included under “Plant polymer degradation”. A red vertical line separates the *Holophaga* and *Terracidiphilus* MAGs. AAs, CAZymes with auxillary activities; GHs, glycosyl hydrolases; and PLs, polysaccharide lyases. Detailed annotation information for putative CAZyme-encoding genes is presented in Table S1.

MAG metabolic reconstructions suggest a clear distinction between *Holophaga* and *Terracidiphilus* in terms of their carbon degradation potential. Carbohydrate active enzyme (CAZyme) profiles (Fig. 2; Table S1) reveal the preponderance of various polysaccharide lyases and glycosyl hydrolases (GHs) in the *Terracidiphilus* genomes, suggesting the relative enrichment of cellulolytic capabilities in this genus compared to *Holophaga* (Fig. 2). The predicted CAZyme functions for *Terracidiphilus* include the ability to degrade pectin, chitin, hemicellulose (including xylan), glucan, pullulan, and starch (Fig. 2; Table S1). While the *Holophaga* MAGs also harbor several hydrolytic enzymes that potentially target starch, glucan, and xylan (Fig. 2), the corresponding GH families appear to be distinct from those found in *Terracidiphilus* (Table S1). Polysaccharide lyases are only annotated in the *Terracidiphilus* genomes, putatively targeting alginate, pectin, hyaluronan, glucuronan, and chondroitin sulfate (Fig. 2). CAZymes with auxiliary activities (AA) appear to have a relatively patchy distribution among the two groups (Fig. 2).

10.1128/msystems.00055-22.1 TABLE S1 Genes encoding putative carbohydrate active enzymes, identified in each MAG using the dbCAN2 server. Download Table S1, PDF file, 0.2 MB. Copyright © 2022 Reji and Zhang. 2022 Reji and Zhang. https://creativecommons.org/licenses/by/4.0/ This content is distributed under the terms of the Creative Commons Attribution 4.0 International license.

Both *Holophaga* and *Terracidiphilus* populations appear to utilize various polysaccharide oligomers; however, notable differences exist in their genomic potential for carbohydrate uptake and utilization. For example, the ability to utilize mannose and trehalose appears to be restricted to certain *Terracidiphilus* lineages, while fucose and fructose utilization potential is patchier within this lineage compared to the *Holophaga* (Fig. 2). Transporter genes identified within each MAG further support the carbon-based niche differentiation between the two genera. *Holophaga* genomes are particularly enriched in membrane transporters (11 to 13% of the total predicted protein coding genes, compared to <7% in the *Terracidiphilus* genomes), most of which are predicted to be various carbohydrate and sugar transporters (Fig. S2). Notably, several of the monosaccharide and oligosaccharide transporters with predicted substrate specificities (e.g., ribose, rhamnose, xylose, and glucan transporters) identified across the *Holophaga* genomes are not present in the relatively divergent *Holophaga* genome, 5AWT5 (Fig. 2). In contrast, *Terracidiphillus* MAGs contain relatively fewer carbohydrate transporters and generally have fewer amino acid transporters with specific predicted substrate affinities (Fig. S2). Both groups, however, appear to be capable of taking up amino acids, oligopeptides, and dipeptides (Fig. S2).

10.1128/msystems.00055-22.4 FIG S2 Transporter genes with substrate predictions identified across the *Holophaga* and *Terracidiphilus* MAGs. The number of copies for each transporter gene has been normalized to the total number of protein coding sequences identified in each genome. Download FIG S2, TIF file, 2.2 MB. Copyright © 2022 Reji and Zhang. 2022 Reji and Zhang. https://creativecommons.org/licenses/by/4.0/ This content is distributed under the terms of the Creative Commons Attribution 4.0 International license.

All *Holophaga* MAGs, except the relatively distant lineage 5AWT5, contain a homolog of AA6 benzoquinone reductase, which is potentially involved in the intracellular reduction of aromatic compounds. In contrast, a putative vanillyl-alcohol oxidase (AA4) is solely found in the 5AWT5 genome (Fig. 2), and it is potentially involved in the oxidoreduction of phenolic compounds derived from lignin degradation. A homolog of the laccase-like polyphenol oxidase previously characterized in Acidobacteria (51) was found in the *Terracidiphilus* MAG 10BWT4. Notably, this MAG was particularly enriched post-oxygenation in the treatment incubations (Fig. 1C).

Consistent with the predicted fermentative lifestyle of the *Holophaga*, various classes of putatively hydrogen-evolving [FeFe] hydrogenases were detected in these genomes (Fig. S3). These include the group A3 and group B fermentative hydrogen-evolving hydrogenases, as well was the group C putatively sensory hydrogenases typically found in anaerobic, fermentative bacteria (52). *Terracidiphilus* MAGs generally lack these [FeFe] hydrogenases and instead encode various [NiFe] hydrogenases (Fig. S3). These include group 1 respiratory hydrogen-oxidizing hydrogenases (Groups 1c and 1d), group 3 putatively bidirectional hydrogenases (3b and 3d), and group 4 respiratory hydrogenases (4b and 4d). A formate dehydrogenase was found in the group 4b hydrogenase gene cluster, suggesting the potential for coupling formate oxidation to proton reduction (Table S2).

10.1128/msystems.00055-22.2 TABLE S2 Hydrogenase classifications, neighborhood comparisons, and inferred functional roles. All analyses are based on Greening et al., 2016 (52). Download Table S2, PDF file, 0.09 MB. Copyright © 2022 Reji and Zhang. 2022 Reji and Zhang. https://creativecommons.org/licenses/by/4.0/ This content is distributed under the terms of the Creative Commons Attribution 4.0 International license.

10.1128/msystems.00055-22.5 FIG S3 Maximum likelihood phylogenetic tree of hydrogenase sequences retrieved from the MAGs (red branches), along with reference sequences (52). Download FIG S3, TIF file, 2.6 MB. Copyright © 2022 Reji and Zhang. 2022 Reji and Zhang. https://creativecommons.org/licenses/by/4.0/ This content is distributed under the terms of the Creative Commons Attribution 4.0 International license.

The capacity for O_2_ respiration is found within both *Terracidiphilus* and *Holophaga*. High-affinity, cbb3-type cytochrome c oxidases are found in some of the *Terracidiphilus* MAGs, while all of the *Holophaga* genomes encode low-affinity, aa3-type cytochrome c oxidases (Fig. 2). The genomes of both genera also encode several genes for dealing with oxidative stress, including superoxide dismutase, catalase peroxidase, and alkyl hydroperoxidase.

### Diazotrophy potential via both canonical and alternative nitrogenase systems.

**FIG 3:**
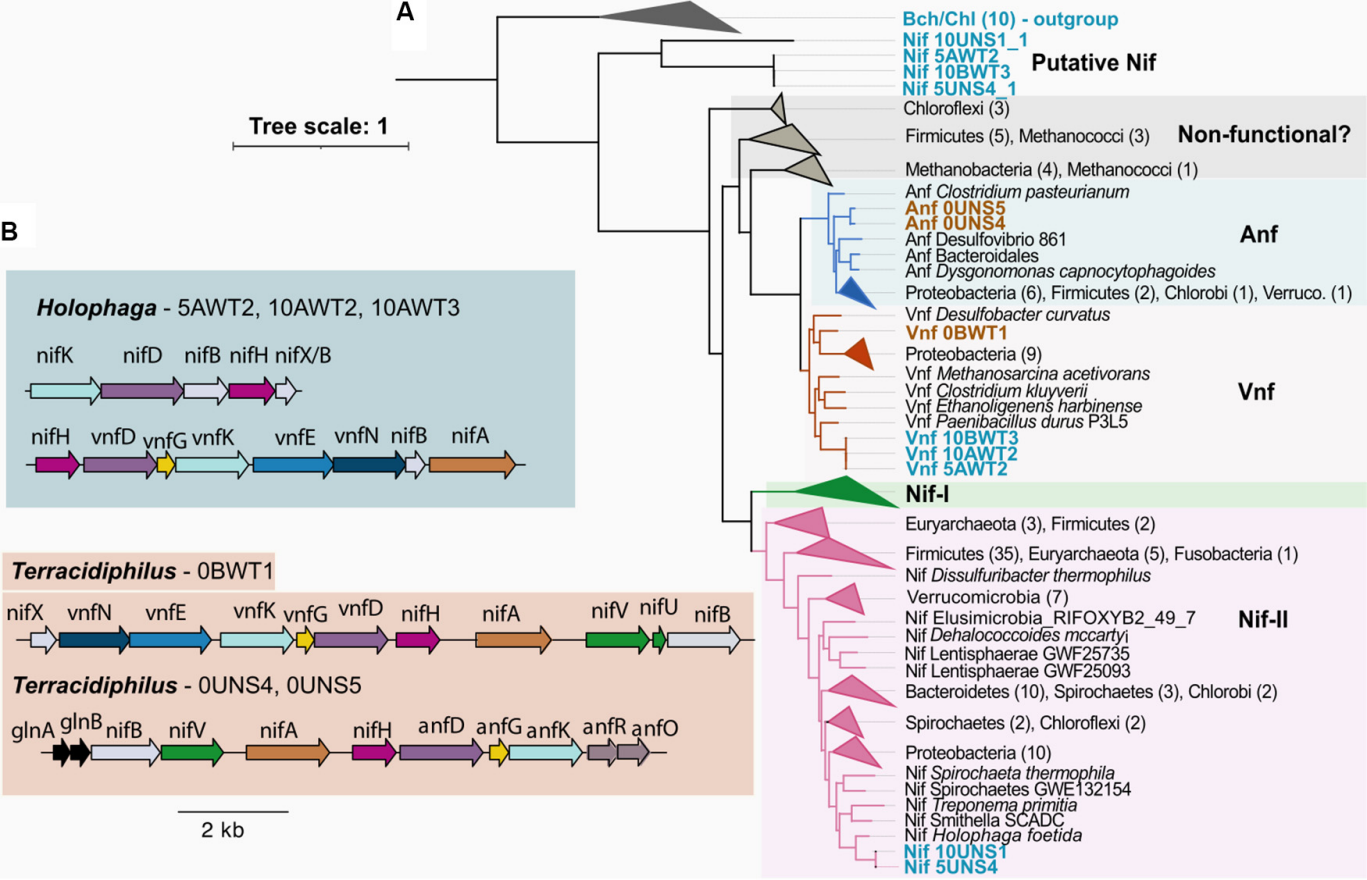
(A) Maximum likelihood phylogenetic tree of a concatenated alignment of the nitrogenase reductase (NifH), and nitrogenase alpha and beta subunits (Nif/Vnf/AnfDK), along with the reference sequences compiled in Garcia et al., 2020 (78). Nif cluster identifications are based on Garcia et al., 2020 (78). (B) Nitrogenase gene neighborhoods are identified in each MAG. Multiple *nifDK* clusters, including the longer *nifKDBHX* cluster that is illustrated here, were present in the *Holophaga* genomes. The alternative nitrogenase clusters each contained the additional G subunit, which is absent in the canonical molybdenum nitrogenase.

The MAG functional classification further indicates the potential for diazotrophy among both the *Terracidiphilus* and the *Holophaga* lineages. The genetic potential for N_2_ fixation is present in 5 of the *Holophaga* genomes (excluding the relatively divergent MAG 5AWT5) and in 3 of the *Terracidiphilus* genomes. Particularly intriguing is the presence of alternative nitrogenase systems in several MAGs, which use a vanadium-iron (V-Fe) or an iron-iron (Fe-Fe) cofactor instead of the canonical molybdenum-iron (Mo-Fe) cofactor. The vanadium nitrogenase (Vnf) sequences encoded in the *Holophaga* genomes cluster nearest to Vnf proteins from Paenibacillus durus, a diazotrophic Firmicute (sharing 80.6%, 80.3%, and 69.4% identities with the *P. durus* VnfH, VnfD, and VnfK sequences, respectively) (Fig. 3A). The Vnf neighborhoods in these MAGs include a dinitrogen reductase (*nifH*), the Vnf-specific *vnfG* subunit alongside the nitrogenase alpha and beta subunits (i.e., *vnfD* and *vnfK*), as well as several genes involved in V-Fe cofactor biosynthesis (*vnfE*, *N*, and *B*) (Fig. 3B). In addition to the V-nitrogenase system, multiple copies of the molybdenum nitrogenase (Mo-Nase) genes, *nifK*, *nifD*, and *nifB*, are found scattered along the Nif-positive *Holophaga* genomes. Only two of these clusters contain a *nifH* gene. Therefore, these two were used in phylogenetic analyses. The longest of the two clusters includes the *nifK*, *nifD*, *nifB*, *nifH*, and *nifX* genes in close proximity. When placed within the Nif/Vnf/AnfHDK protein phylogeny, the *nifH*, *nifD*, and *nifK* sequences from this neighborhood clustered with sequences from Holophaga foetida NifHDK within the Nif-II cluster (Fig. 3B). Interestingly, sequences in the second NifHDK cluster (which does not include any of the additional structural genes) form a deeply rooted clade that is basal to the uncharacterized, putatively nonfunctional Nif sequences reported previously (53) (Fig. 3A). Given the missing cofactor biosynthesis genes and the deeply resolving phylogenetic placement, we suspect that these genes may not produce a functional nitrogenase.

In contrast to the *Holophaga*, the nitrogenase-positive *Terracidiphilus* MAGs are missing the canonical Mo-Nases. This is unusual, as alternative nitrogenases have thus far only been found alongside the Mo-Nase system. Lacking evidence that the Mo-Nase is truly missing in these genomes, we attribute the absence of Mo-Nase to genome fragmentation and incompleteness (Table 1). However, the Anf and Vnf gene clusters in these genomes appear to be sufficient to produce a functional nitrogenase enzyme, as indicated by the presence of various genes required for cofactor biosynthesis (Fig. 3B). The *Terracidiphilus* MAG 0BWT1 harbors Vnf subunits (subunits D, G, and K) in a continuous gene cluster that includes a *nifH* and several genes required for Ve-Fe cofactor assembly. An organizationally similar gene cluster is also found in two other *Terracidiphilus* genomes (0UNS4 and 0UNS5), except that the D, G, and K subunits appear to represent the Fe-only Nase (Anf) gene subunits (Fig. 3A). Thus, our data posits the presence of either V-based or Fe-based diazotrophic potential within *Terracidiphilus*, potentially alongside the canonical Mo-based diazotrophy.

The presence of the alternative nitrogenase systems in *Terracidiphilus* might be linked to the phylogenetic relatedness of these genomes, as the two Anf-positive MAGs (0UNS4 and 0UNS5) are more closely related to each other than to the Vnf-containing 0BWT1 (Fig. 1A). The *Holophaga* genomes also contained Vnf genes; however, the VnfHDK sequences in the *Terracidiphilus* MAG 0BWT1 are phylogenetically distinct from those found within the *Holophaga* genomes; the former forms a sister lineage to proteobacterial Vnf sequences, whereas the *Holophaga* sequences cluster with Firmicute-derived and methanogen-derived Vnf sequences (Fig. 3A). The AnfHDK sequences found in the *Terracidiphilus* genomes form a basal cluster to all of the characterized Anf sequences included in the phylogenetic analysis, except for the more deeply diverging sequences from Clostridium pasteurianum (Fig. 3A).

## DISCUSSION

Microbial community succession patterns following redox shifts in peatlands are important in determining the fate of the peat carbon stock, which accounts for ~20 to 30% of the soil carbon reservoir (54). As demonstrated in (26), the restructuring of microbial communities can have significant impacts on peat biogeochemistry, particularly on the emissions of greenhouse active gases, such as CO_2_ and CH_4_. While O_2_-induced changes in the peat microbial community and the resulting enhancement of CH_4_ fluxes were demonstrated based on 16S rRNA gene abundances and geochemical measurements (26), the mechanisms explaining the differential enrichment of the dominant acidobacterial lineages remained unclear. Our genome-centric approach enabled a detailed resolution of the mechanisms of niche differentiation within this abundant phylum across the O_2_ treatments.

The distinct carbon substrate preferences of *Holophaga* and *Terracidiphilus*, as inferred based on the MAG metabolic reconstructions in this study, point to the vital role that O_2_ levels play in the ecophysiology of these two lineages in peat systems. The apparent enrichment of *Terracidiphilus* lineages within continuously anoxic peat control incubations is likely linked to their metabolic flexibility, which includes the ability to ferment a variety of plant polymers, as evidenced by the diverse CAZyme profile. The Stordalen Mire genomes (21), which constitute the majority of the presently available *Terracidiphilus* reference genomes (Fig. 1A), also harbored the ability to degrade diverse polysaccharides. Moreover, *Acidobacteriales*, which fall within the same class (*Acidobacteriia*) as *Terracidiphilus*, have been found to be abundant in anoxic peat layers in the field (22), suggesting that this lineage may be a key player in peat OM degradation under anoxic conditions. The *Holophaga*, in contrast, appear to have a limited ability to directly degrade large biopolymers (Fig. 2) and may instead benefit from the breakdown products of polysaccharide hydrolysis that can be acquired using the diverse repertoire of transporters found in these genomes (Fig. S2).

The presence of high-affinity and low-affinity terminal oxidases in *Terracidiphilus* and *Holophaga*, respectively, suggests that each lineage may be able to respire O_2_ under aerobic or microaerobic conditions. However, these terminal oxidases, particularly the low-affinity aa3-type, can function in O_2_ scavenging in addition to respiration (5). Indeed, the *Holophaga* isolate *H. foetida* harbors a low-affinity terminal oxidase, despite being described as obligately anaerobic (5). The potential for aerobic respiration in these lineages therefore requires further experimental validation. The various respiratory uptake hydrogenases within *Terracidiphilus* point to a potentially broader range of respiratory strategies for this genus, including the ability to couple proton reduction with energy conservation (group 4d hydrogenases) and respiration involving formate, fumarate, and O_2_ (groups 4b, 1d, and 1c) (Table S2) (52).

**FIG 4:**
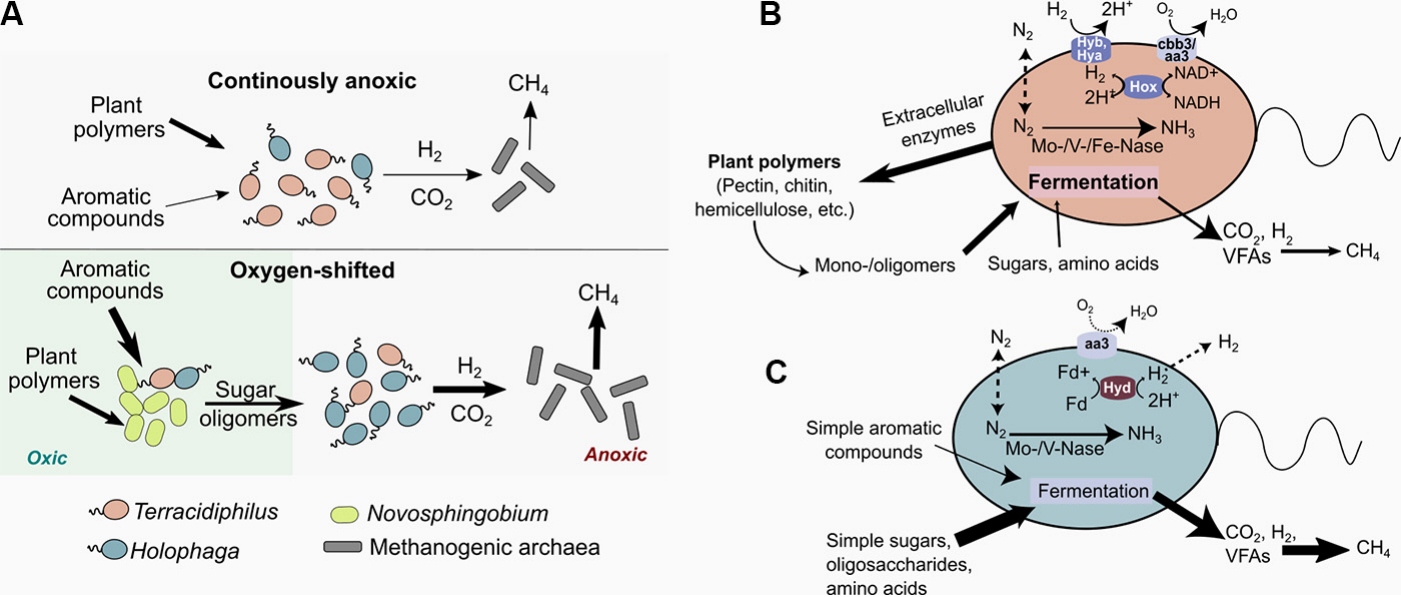
(A) Schematic showing the role of Acidobacteria subpopulations in carbon flow in the peat slurry incubations in response to the O_2_ shift treatments, inferred based on genomic data. In the continuously anoxic incubations, *Terracidiphilus*-mediated carbon flow is predominantly from plant polymers to CO_2_, H_2_, and, ultimately, CH_4_. Exposure to O_2_ stimulates the breakdown of aromatic compounds, predominantly mediated by the aerobic bacterial genus *Novosphingobium*. This promotes the enrichment of *Holophaga*, which are specialized in sugar oligomer degradation. The higher CO_2_ and H_2_ fluxes due to the *Holophaga*-mediated fermentation of sugar oligomers leads to enhanced CH_4_ production in peat pretreated with O_2_. Panels (B) and (C) summarize key metabolic features of the *Terracidiphilus* and *Holophaga* MAGs, respectively. VFAs, volatile fatty acids; Nase, nitrogenase; cbb3, cbb3-type cytochrome c oxidase; aa3, aa3-type cytochrome c oxidase; Mo, molybdenum; V, vanadium; Fe, iron.

The differential enrichment of the Acidobacteria lineages in response to the O_2_ treatment (Fig. 1C) appears to result largely from the O_2_ stimulating the degradation of polyphenolic and other complex plant compounds (26), which provides additional carbon and energy flux for anaerobic carbon conversions, possibly supported by the removal of the phenolic inhibition of hydrolase activity (27, 55). The subsequent increases in the fluxes of carbohydrate oligomers will fuel fermentation, ultimately increasing the substrate flux to methanogenesis (Fig. 4). Metagenomic and 16S rRNA gene data previously indicated an enrichment of phenol oxidase-harboring *Novosphingobium* in peat incubations following oxygenation (26). Wilmoth et al. (26) previously noted that the enhanced consumption of phenolics by these organisms in the presence of O_2_ may have contributed to the enrichment of *Holophaga* during the subsequent anoxic period, as the latter were likely able to utilize the greater fluxes of carbohydrate oligomers, as demonstrated by our analysis, revealing diverse substrate uptake and utilization strategies in the *Holophaga* genomes. We were able to assemble two *Novosphingobium* MAGs that encode a putative polyphenol oxidase, a catechol dioxygenase, and several polyphenol oxidoreductases which putatively target phenolic compounds in the presence of O_2_. In addition, some aromatic compounds may be directly broken down by *Holophaga*, as the genomes encode enzymes with predicted intracellular aromatic degradation activity. The MAG analysis further suggests fermentative H_2_ production by the *Holophaga*, which potentially contributes to the elevated methane production by the hydrogenotrophic methanogen *Methanobacterium*. The *Terracidiphilus*, despite sharing several fermentative metabolic pathways with the *Holophaga*, yet harboring a lower diversity of oligomer transporters, were likely outcompeted by the latter due to the elevated oligosaccharide fluxes during the anoxic phase after the O_2_ exposure period. While the anoxic UNS and BWT layers had distinctly different *Terracidiphilus* lineages at the end of the experiment in the anoxic incubations, any such distinction in the O_2_-exposed peat was largely eclipsed by the enrichment of *Holophaga*, as well as the distant *Terracidiphilus* lineage 10BWT4, across all layers (Fig. 1C). Notably, the enrichment of the relatively divergent lineage 10BWT4 in the O_2_-shifted peat (Fig. 1C) may be explained by the presence of a laccase-like phenol oxidase in this genome (51), which is missing in the remaining *Terracidiphilus* genomes.

### Sphagnum carbon degradation and nitrogen fixation.

Sphagnum-derived organic matter forms the basis of the microbial food chain in sphagnum-dominated peatlands (17). Consistent with recent work identifying Acidobacteria as the major degraders of sphagnum-derived polysaccharides in peat bogs (21, 22), the *Terracidiphilus* GH profiles suggest hydrolytic capabilities that specifically target sphagnum-derived polysaccharides, including rhamnogalacturonan or “sphagnan” (22). Our data indicate a larger role for *Terracidiphilus* lineages in sphagnan breakdown than for *Holophaga*, indicated by the preponderance of rhanogalacturonan-specific CAZymes (both GHs and carbohydrate esterases) in the *Terracidiphilus* genomes (summarized under “pectin” in Fig. 2, with detailed annotations presented in Table S1). Sphagnan degradation releases various monosaccharides, such as arabinose, mannose, and rhamnose, and both *Holophaga* and *Terracidiphilus* MAGs encode degradative enzymes that putatively target these compounds (Fig. 2).

The microbial degradation of the relatively nutrient-poor sphagnum litter yields little nitrogen (17), and the external input of nitrogen into acidic peatlands is primarily from atmospheric deposition (47). Biological nitrogen fixation in sphagnum-dominated peat systems has been attributed to sphagnum-associated methanotrophs, with potential contributions from Cyanobacteria and heterotrophic bacteria (38, 39, 47, 56, and references therein). The relative importance of methanotrophs as nitrogen fixers in peat systems has been challenged in recent years based on observations suggesting the lack of diazotrophy stimulation by higher levels of methane (38). At least in surface peat, diazotrophy appears to be mediated by Alphaproteobacteria (38, 57). To the best of our knowledge, diazotrophy in peatlands has yet to be linked to Acidobacteria. Even more intriguing is the presence of alternative nitrogenase systems in the MAGs described here. Alternative nitrogenases (alt-Nases) containing vanadium (V) or iron (Fe) only are typically used as backup systems when the molybdenum (Mo) supporting the more comprehensively efficient Mo-Nase system is limited (58, 59). Biogeochemical processes, including BNF, in peatlands can be affected by trace metal limitation (37). A previous study examining the nitrogenase diversity and activity in peat soils reported that the alt-Nases comprise <5% of the overall nitrogenase abundance across soil depths, although the Mo concentrations in surface peat were over 3-fold lower than the levels of V (38). The alt-Nase sequences previously recovered in (38) clustered with *vnfD/anfD* sequences from Proteobacteria, while those found in our MAGs were phylogenetically most similar to Firmicute alt-Nases, suggesting potential lateral gene acquisition (Fig. 3A). Given the notably high relative abundance of Acidobacteria observed in peat systems, their potential for contributing to BNF and the trace metal conditions supporting those contributions in these habitats warrant further investigation.

### Conclusions.

Understanding the mechanistic underpinnings of peat carbon degradation to CO_2_ and CH_4_, specifically the controls imparted by microbial functional guilds, is critical for climate change mitigation efforts. A genome-centric approach that complements conventional amplicon-based analyses is particularly instrumental in constraining population-specific or lineage-specific metabolic strategies that ultimately govern carbon fluxes in complex systems. Our genome-centered metagenomic analysis of peat Acidobacteria supports the previously identified roles of these microbes as key carbon degraders and also provides additional phylogenetic and metabolic resolution on lineage-specific contributions to peat carbon transformations under varying redox regimes. We find that different Acidobacteria populations are differentially influenced by the redox oscillations resulting from O_2_ fluctuations. The metabolically versatile *Terracidiphilus* dominate under anoxic conditions, while O_2_ exposure leads to the enrichment of *Holophaga*, which has a more limited polysaccharide degradation potential but an enhanced potential for oligosaccharide utilization. Consistent with previously outlined microbially-driven carbon flows (26), under elevated oligosaccharide fluxes resulting from the O_2_-stimulated breakdown of complex aromatic and carbohydrate-bearing biopolymers in peat, the *Holophaga* appear to outcompete *Terracidiphilus*, ultimately leading to an enhanced substrate flow toward methanogenesis. Importantly, our data also suggest a previously unrecognized role of Acidobacteria in peat nitrogen cycling, as the potential for diazotrophy was found in both the *Holophaga* and the *Terracidiphilus* genomes. Particularly intriguing is the presence of divergent forms of both canonical and alternative nitrogenase systems in these lineages. Our results map out a more phylogenetically and metabolically resolved view of the ecophysiology of peat Acidobacteria in relation to O_2_-driven redox fluctuations and invite further investigation into their roles in carbon and nitrogen transformations in peat and other soil systems that are sensitive to global change.

## MATERIALS AND METHODS

### Sample collection, incubation set up, and metagenome sequencing.

Detailed descriptions of sample collection, processing, and sequencing are provided in (26). Briefly, peat samples of three distinct layers (i.e., unsaturated, below water table, and above water table) collected from the Ward Reservation (Andover, MA, USA) were ground into layer-specific slurries with filtered porewater (10% vol/vol) while continuously being flushed with nitrogen gas (N_2_). For each layer type, 90 mL of slurry material were added to 160 mL serum vials that were sealed with butyl rubber stoppers and aluminum seals. The vials were either incubated under continuously anoxic conditions for 232 days, or exposed to O_2_ for 98 days, followed by anoxic incubation for 134 days. The O_2_ pretreatment involved either a 5% or 10% vol/vol addition of O_2_ in the incubation headspace via flushing with a mixture of analytical grade air and 100% N_2_. Anoxic conditions were established by flushing the serum vials that contained the peat slurries with 100% N_2_ gas. The samples for molecular analyses were subsampled from the incubation slurries using a sterile needle attached to a Leur-lock syringe, which was flushed with 100% N_2_ gas prior to sampling to preserve the anoxic conditions. The slurry samples were collected on days 21, 98, 126, 198, and 232, which we sequentially denote as time points 1 to 5 (T1 to T5). Total nucleic acids from the slurry subsamples were extracted with the RNeasy PowerSoil Total RNA Kit (Qiagen). Purified DNA was eluted with the PowerSoil DNA Elution Kit (Qiagen) and used for metagenomic sequencing on the Illumina HiSeq platform at Molecular Research LP (MR DNA). The RNA extracts were not used in sequencing due to poor quality.

### Metagenome assembly, genome binning, and taxonomic classification.

Raw sequence libraries deposited in the Sequence Read Archive (accession no. PRJNA551662), as indicated in (26), were used for assembly and binning. Paired-end reads were quality-filtered by using Trimmomatic (v0.39) (60). A sliding window of size 10 was used to remove bases with a Phred quality score of <28. Reads that were at least 75 bp long were retained for downstream processing. Read quality metrics obtained via FastQC (v0.11.9) (61) were used to guide the quality-filtering step. Assembly was performed using MEGAHIT (v1.2.9) (62, 63), using the k-mers 21, 33, 55, 77, 99, and 127. The read libraries originating from each peat layer at the final time point were coassembled. DNA from earlier time points were pooled for sequencing owing to low yield (26), and the resulting read libraries were assembled individually. Contigs longer than 2,000 bp were subsequently binned using MetaBAT2 (v1.12.1) (64) and MaxBin2 (v2.2.7) (65, 66). The bin refinement module in metaWRAP (v1.2) (67) was used to refine the assembled bins, which involved reassembly using SPAdes (v3.13.0) (68) to improve the assembly quality. Genomes were further refined by removing outlier contigs identified based on tetranuleotide frequencies, GC content, known contaminants, and clade markers, using default parameters for each module in the MAGpurify package (v2.1.2) (69). CheckM (v1.0.13) (70) was used to assess genome completion and redundancy, and those with an estimated completeness of ≥70% and a contamination of <10% were retained for downstream analyses. The Genome Taxonomy Database toolkit (GTDB-tk) Release 05-RS95 (71) was used to obtain taxonomic classifications for the assembled genomes.

### Genome annotations and functional inference.

Prodigal (v2.6.3) (72) was used to predict protein-coding genes, followed by initial functional annotations using Prokka (v1.14.5) (73). GhostKOALA (74) and eggNOG-mapper (75, 76) were used to obtain KO annotations, which were then used for metabolic reconstructions via the “Reconstruct Pathway” tool in KEGG Mapper (77). SEED annotations were obtained from the online Rapid Annotation using Subsystem Technology (RAST) server (78). Annotations for genes of interest were compared across the various annotation tool outputs and double-checked by BLASTP searches against the NCBI nonredundant protein, RefSeq, and UniprotKB/Swiss-port databases. Neighborhood comparisons were also employed to improve confidence in annotations (e.g., nitrogenases). TransportDB (v2.0) (79) was used to predict transporters and their putative substrates. CAZyme annotations were obtained via the dbCAN meta server (80), using amino acid sequences for each genome as queries. The dbCAN runs were conducted using both HMMER and DIAMOND searches with default parameters, and the results were interpreted using the CAZy database (81). CAZyme annotations agreed upon by both HMMER and DIAMOND were retained for downstream analysis.

### Phylogenetic analyses.

Reference Acidobacteria genomes were retrieved from the NCBI GenBank database, based on the Acidobacteria reference tree in GTDB R202 (82). Conserved ribosomal marker genes were extracted from both the reference genomes and the MAGs using the phylogenomics module in Anvi’o (v7) (83). The following marker genes were included in the analysis: ribosomal proteins S12_S23, L1, L13, L14, L16, L17, L18p, L19, L2, L20, L21p, L22, L23, L27, L27A, L28, L29, L3, L32p, L35p, L4, L5, L6, L9_C, S10, S11, S13, S15, S16, S17, S19, S2, S20p, S3_C, S6, S7, S8, and S9. A concatenated alignment was generated using MUSCLE (v3.8.1551) (84), and trimAL (v1.4.rev15) (85) was used to trim the alignment using the flags “-gt 0.80” and “-st 0.001”. The trimmed alignment was used as the input in IQ-TREE (v2.1.3) (86) to generate a maximum likelihood phylogenomic tree, using 1,000 bootstrap replicates with ultrafast bootstrap approximation (87). The tree was visualized in FigTree (http://tree.bio.ed.ac.uk/software/figtree/) and modified in Inkscape (https://inkscape.org) to highlight clusters of interest.

The amino acid sequences compiled in (53) were used as the references for a nitrogenase phylogenetic analysis. Sequences of nitrogenase reductase (NifH), along with nitrogenase alpha and beta subunits (i.e., NifD and NifK, respectively), retrieved from the MAGs, were aligned with corresponding reference sequences using MAFFT (v7.475) (88). The three individual alignments were concatenated and used for phylogenetic inference using IQ-TREE (v2.1.3) (86) with 1,000 bootstrap replicates. Phylogenetic clusters were identified based on the classification presented in (53).

Hydrogenase homologs were identified in each genome using a DIAMOND BLASTP search (89) of translated sequences against a protein database generated by using the reference hydrogenase sequences compiled in (52). Hits with a minimum E value of 1E−100 were retained for downstream analyses. Sequences were aligned using MAFFT (v7.475) (88) and trimmed using trimAL (v1.4.rev15) (85) with the flags “-gt 0.8” and “-cons 60”. Finally, IQ-TREE (v2.1.3) (86) was used to generate a maximum likelihood tree using the trimmed alignment. The tree was visualized and edited in iTol (v6) (90).

### Data availability.

The metagenome sequence libraries analyzed in this project are available in the NCBI Sequence Read Archive (SRA) under the BioProject accession number PRJNA551662. The FASTA files of the genome sequences assembled here have been deposited in Figshare: https://figshare.com/s/4424c7321d549bdd6704.
