# Supplementary figures and images for: Genome-Resolved Metagenomics Informs the Functional Ecology of Uncultured Acidobacteria in Redox Oscillated Sphagnum Peat
Source: mSystems. 2022 Aug 29;7(5):e00055-22. doi: 10.1128/msystems.00055-22 (PMC9599518; doi:10.1128/msystems.00055-22)

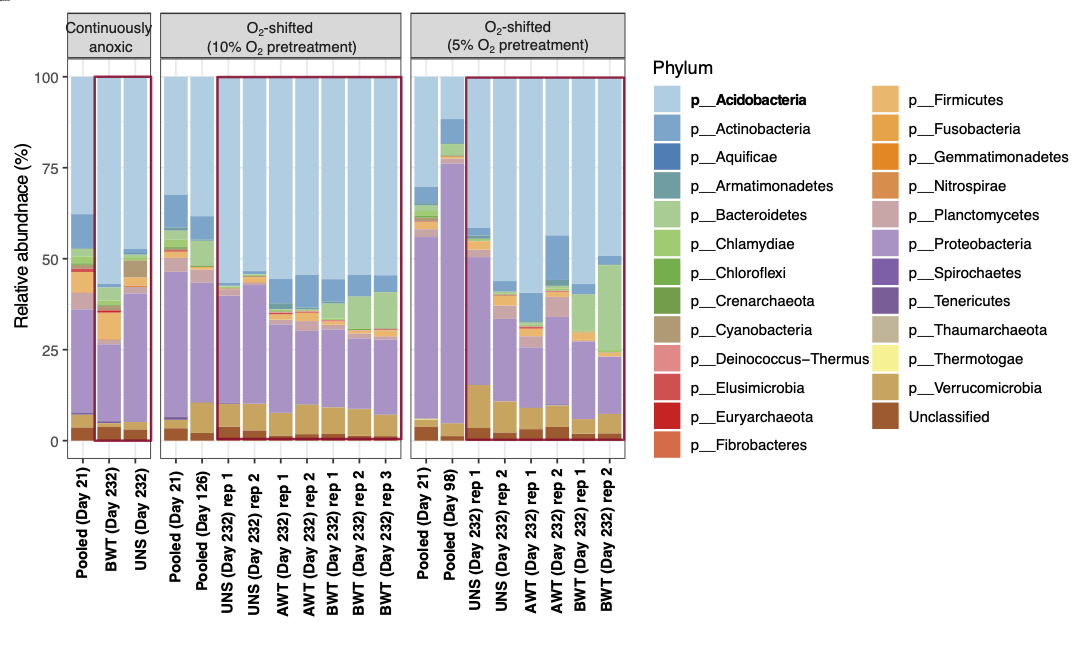

Supplement: FIG S1 [file msystems.00055-22-s0001.tif]

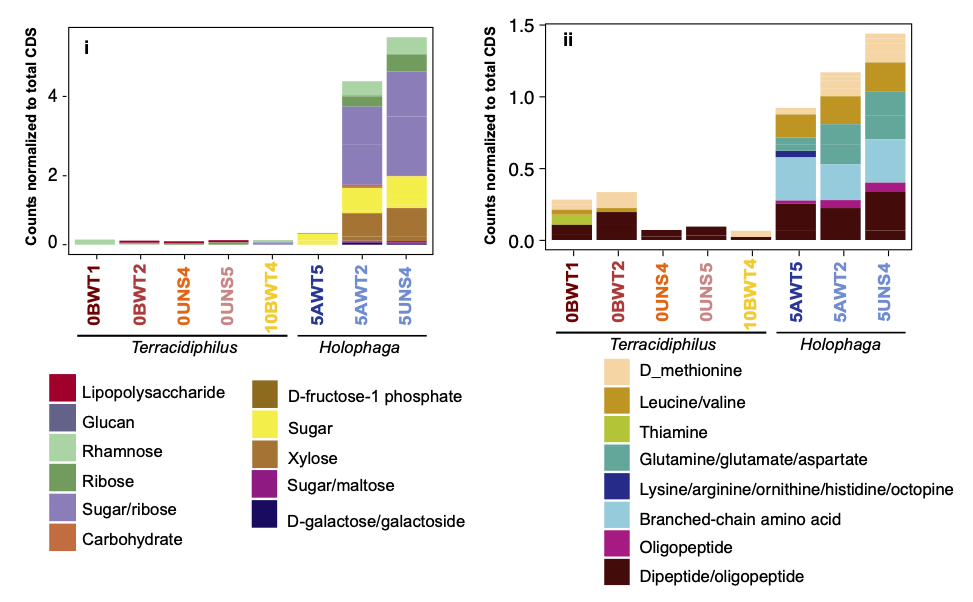

Supplement: FIG S2 [file msystems.00055-22-s0002.tif]

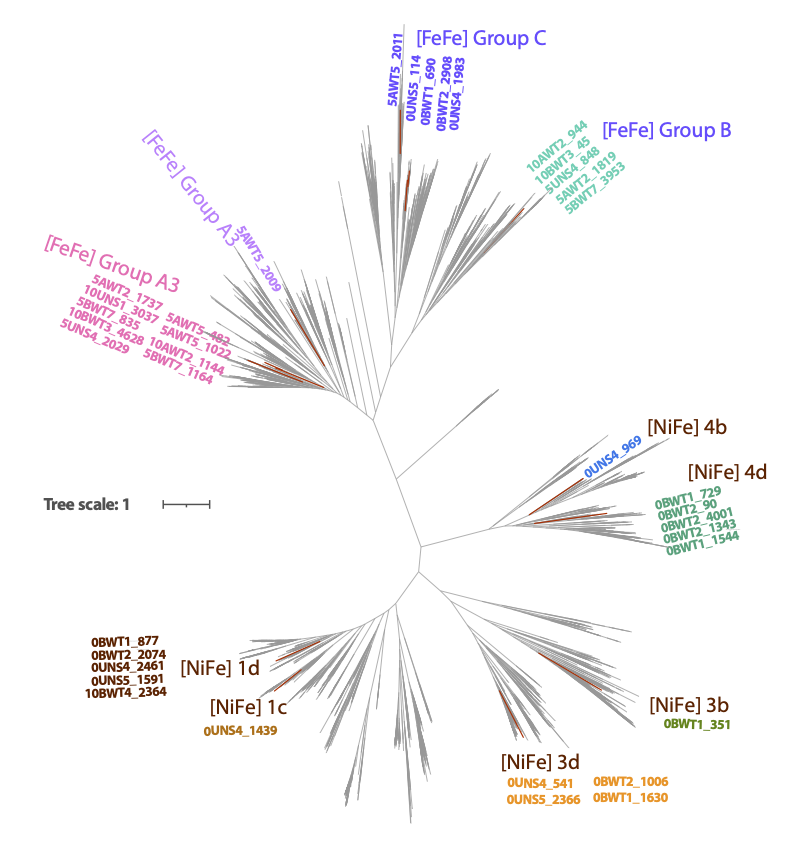

Supplement: FIG S3 [file msystems.00055-22-s0003.tif]
