# Supplementary material for: Genome-Resolved Metagenomics Informs the Functional Ecology of Uncultured Acidobacteria in Redox Oscillated Sphagnum Peat
Source: mSystems. 2022 Aug 29;7(5):e00055-22. doi: 10.1128/msystems.00055-22 (PMC9599518; doi:10.1128/msystems.00055-22)
Supplement: TABLE S1 [file msystems.00055-22-s0004.pdf]

Table S1: Genes encoding putative carbohydrate active enzymes identified in each MAG using the dbCAN2 server.

Gene_ID	MAG	Gene numbe	Genus	HMMER	DIAMOND	Signalp	Putative substrate/function
0BWT2_2321	0BWT2	2321	Terracidiphlus	PL5(20-334)	PL5	Y(1-20)	Alginate
0BWT2_3771	0BWT2	3771	Terracidiphlus	PL7_4(51-263)	PL7_4	Y(1-40)	Alginate
0BWT1_3052	0BWT1	3052	Terracidiphlus	GH127(344-570)	CBM66	N	beta-L-arabinofuranose cleavage
10BWT4_1492	10BWT4	1492	Terracidiphlus	GH127(73-596)	GH127	Y(1-27)	beta-L-arabinofuranose cleavage
0BWT2_2515	0BWT2	2515	Terracidiphlus	GH94(2-614)	GH94	N	cellobiose, cellodextrin, chitobiose
0BWT1_2601	0BWT1	2601	Terracidiphlus	GH94(2-808)	GH94	N	cellobiose, cellodextrin, chitobiose
0BWT1_980	0BWT1	980	Terracidiphlus	GH3(102-304)	GH3	N	Cellulose
0UNS5_1116	0UNS5	1116	Terracidiphlus	GH3(108-329)	GH3	Y(1-26)	Cellulose
10BWT4_21	10BWT4	21	Terracidiphlus	GH3(109-343)	GH3	N	Cellulose
0BWT2_1820	0BWT2	1820	Terracidiphlus	GH3(109-343)	GH3	N	Cellulose
5UNS4_1416	5UNS4	1416	Holophaga	GH3(111-319)	GH3	Y(1-56)	Cellulose
0BWT2_1889	0BWT2	1889	Terracidiphlus	GH3(111-331)	GH3	Y(1-29)	Cellulose
0UNS4_790	0UNS4	790	Terracidiphlus	GH3(114-346)	GH3	Y(1-25)	Cellulose
0BWT2_1615	0BWT2	1615	Terracidiphlus	GH3(121-343)	GH3	N	Cellulose
10BWT4_511	10BWT4	511	Terracidiphlus	GH3(123-356)	GH3	Y(1-35)	Cellulose
0UNS4_114	0UNS4	114	Terracidiphlus	GH3(125-333)	GH3	N	Cellulose
0BWT2_949	0BWT2	949	Terracidiphlus	GH3(126-350)	GH3	Y(1-32)	Cellulose
0BWT1_2412	0BWT1	2412	Terracidiphlus	GH3(136-346)	GH3	N	Cellulose
10BWT4_2846	10BWT4	2846	Terracidiphlus	GH3(137-361)	GH3	Y(1-37)	Cellulose
0BWT2_1055	0BWT2	1055	Terracidiphlus	GH3(14-255)	GH3	N	Cellulose
10BWT4_578	10BWT4	578	Terracidiphlus	GH3(144-366)	GH3	Y(1-41)	Cellulose
0UNS4_1507	0UNS4	1507	Terracidiphlus	GH3(154-376)	GH3	N	Cellulose
10BWT4_2553	10BWT4	2553	Terracidiphlus	GH3(16-257)	GH3	N	Cellulose
0BWT1_474	0BWT1	474	Terracidiphlus	GH3(163-386)	GH3	Y(1-50)	Cellulose
0UNS5_567	0UNS5	567	Terracidiphlus	GH3(17-227)	GH3	N	Cellulose
10BWT4_1489	10BWT4	1489	Terracidiphlus	GH3(35-203)	GH3	N	Cellulose
0BWT2_1414	0BWT2	1414	Terracidiphlus	GH3(54-263)	GH3	N	Cellulose
0BWT1_2718	0BWT1	2718	Terracidiphlus	GH3(61-302)	GH3	Y(1-21)	Cellulose
0BWT2_1796	0BWT2	1796	Terracidiphlus	GH3(64-304)	GH3	Y(1-22)	Cellulose
10BWT4_1044	10BWT4	1044	Terracidiphlus	GH3(64-305)	GH3	Y(1-23)	Cellulose
0BWT1_1841	0BWT1	1841	Terracidiphlus	GH3(65-272)	GH3	Y(1-27)	Cellulose
0BWT2_572	0BWT2	572	Terracidiphlus	GH3(65-287)	GH3	N	Cellulose
0BWT1_3406	0BWT1	3406	Terracidiphlus	GH3(66-306)	GH3	Y(1-25)	Cellulose
0BWT2_1616	0BWT2	1616	Terracidiphlus	GH3(68-308)	GH3	Y(1-30)	Cellulose
0UNS5_76	0UNS5	76	Terracidiphlus	GH3(69-291)	GH3	N	Cellulose
0BWT2_1293	0BWT2	1293	Terracidiphlus	GH3(69-309)	GH3	Y(1-32)	Cellulose
0UNS4_1244	0UNS4	1244	Terracidiphlus	GH3(72-312)	GH3	Y(1-39)	Cellulose
0UNS5_816	0UNS5	816	Terracidiphlus	GH3(72-312)	GH3	Y(1-27)	Cellulose
0BWT1_172	0BWT1	172	Terracidiphlus	GH3(73-281)	GH3	Y(1-31)	Cellulose
0BWT2_1370	0BWT2	1370	Terracidiphlus	GH3(75-281)	GH3	Y(1-34)	Cellulose
0BWT2_943	0BWT2	943	Terracidiphlus	GH3(77-290)	GH3	Y(1-36)	Cellulose
0BWT2_645	0BWT2	645	Terracidiphlus	GH3(77-319)	GH3	Y(1-29)	Cellulose
0BWT2_3044	0BWT2	3044	Terracidiphlus	GH3(79-311)+CBM	CBM6+GH3	N	Cellulose
0UNS4_1450	0UNS4	1450	Terracidiphlus	GH3(82-292)	GH3	Y(1-21)	Cellulose
0UNS5_209	0UNS5	209	Terracidiphlus	GH3(86-327)	GH3	Y(1-22)	Cellulose
0UNS4_2254	0UNS4	2254	Terracidiphlus	GH3(92-333)	GH3	Y(1-22)	Cellulose
0BWT2_715	0BWT2	715	Terracidiphlus	GH3(95-308)	GH3	N	Cellulose
0BWT1_1977	0BWT1	1977	Terracidiphlus	GH3(96-304)	GH3	Y(1-40)	Cellulose
0BWT2_329	0BWT2	329	Terracidiphlus	GH5_13(56-330)	GH5_13	Y(1-28)	cellulose
0BWT1_3047	0BWT1	3047	Terracidiphlus	GH5_13(61-335)	GH5_13	Y(1-32)	cellulose
0BWT1_3463	0BWT1	3463	Terracidiphlus	GH5_24(71-379)	GH5_24	N	cellulose
0BWT2_1644	0BWT2	1644	Terracidiphlus	CE7(97-262)	CE7	Y(1-25)	Cephalosporin-C, xylan and xylo-oligosaccharides
0UNS4_1340	0UNS4	1340	Terracidiphlus	GH18(188-444)+C	CE4+GH18+GT	N	Chitin
0BWT2_2241	0BWT2	2241	Terracidiphlus	GH18(192-426)+C	CE4+GH18+GT	N	Chitin
0BWT1_1999	0BWT1	1999	Terracidiphlus	GH18(195-430)+C	CE4+GH18+GT	N	Chitin
0BWT2_1329	0BWT2	1329	Terracidiphlus	GH18(31-361)	GH18	Y(1-23)	Chitin
0UNS4_1586	0UNS4	1586	Terracidiphlus	GH18(73-333)	GH18	Y(1-19)	Chitin
0UNS5_1565	0UNS5	1565	Terracidiphlus	GH18(89-334)	GH18	Y(1-21)	Chitin
0UNS5_2606	0UNS5	2606	Terracidiphlus	GH18(92-351)+CE	CE4+GH18+GT	N	Chitin
10BWT4_2430	10BWT4	2430	Terracidiphlus	PL37(1-386)	PL37	N	chondroitin sulfate
0UNS4_1428	0UNS4	1428	Terracidiphlus	CBM66(53-212)	CBM66	N	Fructan
0BWT1_3027	0BWT1	3027	Terracidiphlus	CBM66(56-213)	CBM66	Y(1-32)	Fructan
0BWT1_451	0BWT1	451	Terracidiphlus	CBM66(56-215)	CBM66	Y(1-35)	Fructan
0UNS5_62	0UNS5	62	Terracidiphlus	CBM66(56-216)	CBM66	Y(1-34)	Fructan
0BWT1_760	0BWT1	760	Terracidiphlus	GH29(105-483)	GH29	Y(1-29)	fucose cleavage
0UNS5_2838	0UNS5	2838	Terracidiphlus	GH29(11-305)	GH29	N	fucose cleavage

0UNS4_2375	0UNS4	2375	Terracidiphlus	GH29(24-396)	GH29	Y(1-28)	fucose cleavage
0UNS5_2786	0UNS5	2786	Terracidiphlus	GH29(3-319)	GH29	N	fucose cleavage
0UNS4_2059	0UNS4	2059	Terracidiphlus	GH29(30-329)	GH29	Y(1-35)	fucose cleavage
0BWT1_3402	0BWT1	3402	Terracidiphlus	GH29(33-328)	GH29	Y(1-32)	fucose cleavage
0BWT1_1285	0BWT1	1285	Terracidiphlus	GH29(33-332)	GH29	Y(1-24)	fucose cleavage
0BWT1_2068	0BWT1	2068	Terracidiphlus	GH29(33-398)	GH29	N	fucose cleavage
0UNS4_2477	0UNS4	2477	Terracidiphlus	GH29(34-388)	GH29	N	fucose cleavage
0BWT2_1957	0BWT2	1957	Terracidiphlus	GH29(35-350)	GH29	Y(1-33)	fucose cleavage
10BWT4_3138	10BWT4	3138	Terracidiphlus	GH29(37-410)	GH29	Y(1-21)	fucose cleavage
10BWT4_2815	10BWT4	2815	Terracidiphlus	GH29(37-416)	GH29	Y(1-29)	fucose cleavage
10BWT4_3093	10BWT4	3093	Terracidiphlus	GH29(38-353)	GH29	N	fucose cleavage
0BWT2_4283	0BWT2	4283	Terracidiphlus	GH29(40-350)	GH29	N	fucose cleavage
0BWT2_702	0BWT2	702	Terracidiphlus	GH29(43-400)	GH29	Y(1-28)	fucose cleavage
0UNS5_189	0UNS5	189	Terracidiphlus	GH29(55-407)	GH29	Y(1-36)	fucose cleavage
0BWT1_2073	0BWT1	2073	Terracidiphlus	GH29(59-467)	GH29	Y(1-32)	fucose cleavage
10BWT4_3236	10BWT4	3236	Terracidiphlus	GH95(1-523)	GH95	N	fucose cleavage
0BWT2_890	0BWT2	890	Terracidiphlus	GH95(248-970)	GH95	Y(1-29)	fucose cleavage
0BWT1_2750	0BWT1	2750	Terracidiphlus	GH95(33-778)	GH95	Y(1-21)	fucose cleavage
0BWT2_1954	0BWT2	1954	Terracidiphlus	GH95(35-674)	CBM32+GH95	N	fucose cleavage
0BWT2_1951	0BWT2	1951	Terracidiphlus	GH95(38-433)	GH95	Y(1-35)	fucose cleavage
0BWT1_635	0BWT1	635	Terracidiphlus	GH95(44-833)	GH95	N	fucose cleavage
10BWT4_3487	10BWT4	3487	Terracidiphlus	GH36(12-580)	GH36	N	galactose cleavage
0BWT2_2396	0BWT2	2396	Terracidiphlus	GH36(33-720)	GH36	Y(1-21)	galactose cleavage
0BWT1_2749	0BWT1	2749	Terracidiphlus	GH36(69-735)	GH36	N	galactose cleavage
10BWT4_3032	10BWT4	3032	Terracidiphlus	GH35(2-229)	GH35	N	galactose cleavage
0UNS5_63	0UNS5	63	Terracidiphlus	GH35(37-214)	GH35	Y(1-23)	galactose cleavage
0UNS5_2468	0UNS5	2468	Terracidiphlus	GH35(38-351)	GH35	Y(1-26)	galactose cleavage
0UNS4_410	0UNS4	410	Terracidiphlus	GH35(40-353)	GH35	Y(1-28)	galactose cleavage
0BWT2_3667	0BWT2	3667	Terracidiphlus	GH35(41-248)	GH35	Y(1-27)	galactose cleavage
0BWT1_3559	0BWT1	3559	Terracidiphlus	GH35(44-357)	GH35	Y(1-30)	galactose cleavage
0UNS5_1409	0UNS5	1409	Terracidiphlus	GH35(47-361)	GH35	Y(1-39)	galactose cleavage
0UNS4_502	0UNS4	502	Terracidiphlus	GH35(50-364)	GH35	Y(1-32)	galactose cleavage
0BWT1_3399	0BWT1	3399	Terracidiphlus	GH35(50-366)	GH35	N	galactose cleavage
10BWT4_2741	10BWT4	2741	Terracidiphlus	GH35(51-364)	GH35	Y(1-26)	galactose cleavage
0BWT1_1461	0BWT1	1461	Terracidiphlus	GH35(57-344)	GH35	Y(1-32)	galactose cleavage
0BWT1_3076	0BWT1	3076	Terracidiphlus	GH35(60-251)	GH35	N	galactose cleavage
0BWT1_1142	0BWT1	1142	Terracidiphlus	GH35(62-407)	GH35	Y(1-20)	galactose cleavage
0BWT2_1608	0BWT2	1608	Terracidiphlus	GH35(70-383)	GH35	N	galactose cleavage
0UNS4_1124	0UNS4	1124	Terracidiphlus	GH35(70-414)	GH35	N	galactose cleavage
0BWT1_2039	0BWT1	2039	Terracidiphlus	GH42(54-418)	GH42	Y(1-33)	galactose/arabinose/fucose cleavage
10BWT4_2268	10BWT4	2268	Terracidiphlus	GH42(57-419)	GH42	N	galactose/arabinose/fucose cleavage
10AWT2_1087	10AWT2	1087	Holophaga	GH42(6-389)	GH42	N	galactose/arabinose/fucose cleavage
10BWT3_721	10BWT3	721	Holophaga	GH42(6-389)	GH42	N	galactose/arabinose/fucose cleavage
5AWT2_2084	5AWT2	2084	Holophaga	GH42(6-389)	GH42	N	galactose/arabinose/fucose cleavage
5BWT7_523	5BWT7	523	Holophaga	GH42(6-389)	GH42	N	galactose/arabinose/fucose cleavage
0BWT1_2954	0BWT1	2954	Terracidiphlus	GH42(79-451)	GH42	N	galactose/arabinose/fucose cleavage
0BWT1_1122	0BWT1	1122	Terracidiphlus	GH42(85-451)	GH42	N	galactose/arabinose/fucose cleavage
0BWT1_1452	0BWT1	1452	Terracidiphlus	GH42(92-452)	GH42	N	galactose/arabinose/fucose cleavage
10BWT4_3135	10BWT4	3135	Terracidiphlus	GH2(10-360)	GH2	N	galactose, mannose, glucuronic acid, arabinofuranose cleavage
0BWT1_2038	0BWT1	2038	Terracidiphlus	GH2(102-540)	GH2	N	galactose, mannose, glucuronic acid, arabinofuranose cleavage
10BWT4_2210	10BWT4	2210	Terracidiphlus	GH2(19-346)	GH2	N	galactose, mannose, glucuronic acid, arabinofuranose cleavage
0BWT1_2729	0BWT1	2729	Terracidiphlus	GH2(22-755)	GH2	Y(1-20)	galactose, mannose, glucuronic acid, arabinofuranose cleavage
0BWT2_575	0BWT2	575	Terracidiphlus	GH2(225-949)	GH2	N	galactose, mannose, glucuronic acid, arabinofuranose cleavage
0BWT1_960	0BWT1	960	Terracidiphlus	GH2(23-690)	GH2	Y(1-22)	galactose, mannose, glucuronic acid, arabinofuranose cleavage
0UNS4_484	0UNS4	484	Terracidiphlus	GH2(25-474)	GH2	Y(1-27)	galactose, mannose, glucuronic acid, arabinofuranose cleavage
0BWT2_1913	0BWT2	1913	Terracidiphlus	GH2(25-742)	GH2	Y(1-27)	galactose, mannose, glucuronic acid, arabinofuranose cleavage
0UNS5_221	0UNS5	221	Terracidiphlus	GH2(27-544)	GH2	Y(1-27)	galactose, mannose, glucuronic acid, arabinofuranose cleavage
0BWT2_2487	0BWT2	2487	Terracidiphlus	GH2(27-594)	GH2	Y(1-26)	galactose, mannose, glucuronic acid, arabinofuranose cleavage
0BWT2_708	0BWT2	708	Terracidiphlus	GH2(28-499)	GH2	Y(1-28)	galactose, mannose, glucuronic acid, arabinofuranose cleavage
0UNS5_436	0UNS5	436	Terracidiphlus	GH2(28-737)	GH2	Y(1-24)	galactose, mannose, glucuronic acid, arabinofuranose cleavage
0UNS4_2098	0UNS4	2098	Terracidiphlus	GH2(29-565)	GH2	Y(1-28)	galactose, mannose, glucuronic acid, arabinofuranose cleavage
0UNS5_135	0UNS5	135	Terracidiphlus	GH2(29-565)	GH2	Y(1-28)	galactose, mannose, glucuronic acid, arabinofuranose cleavage
0BWT1_1753	0BWT1	1753	Terracidiphlus	GH2(30-742)	GH2	Y(1-29)	galactose, mannose, glucuronic acid, arabinofuranose cleavage
0BWT2_1974	0BWT2	1974	Terracidiphlus	GH2(31-718)	GH2	N	galactose, mannose, glucuronic acid, arabinofuranose cleavage
0BWT2_2711	0BWT2	2711	Terracidiphlus	GH2(32-513)	GH2	Y(1-24)	galactose, mannose, glucuronic acid, arabinofuranose cleavage
0UNS5_592	0UNS5	592	Terracidiphlus	GH2(32-714)	GH2	Y(1-28)	galactose, mannose, glucuronic acid, arabinofuranose cleavage
0UNS4_1804	0UNS4	1804	Terracidiphlus	GH2(32-715)	GH2	Y(1-28)	galactose, mannose, glucuronic acid, arabinofuranose cleavage
0UNS4_1616	0UNS4	1616	Terracidiphlus	GH2(32-747)	GH2	Y(1-24)	galactose, mannose, glucuronic acid, arabinofuranose cleavage

0BWT2_2853	0BWT2	2853	Terracidiphlus	GH2(33-528)	GH2	Y(1-24)	galactose, mannose, glucuronic acid, arabinofuranose cleavage
0BWT1_3401	0BWT1	3401	Terracidiphlus	GH2(33-716)	GH2	Y(1-30)	galactose, mannose, glucuronic acid, arabinofuranose cleavage
10BWT4_3329	10BWT4	3329	Terracidiphlus	GH2(37-650)	GH2	Y(1-30)	galactose, mannose, glucuronic acid, arabinofuranose cleavage
10BWT4_129	10BWT4	129	Terracidiphlus	GH2(38-592)	GH2	Y(1-38)	galactose, mannose, glucuronic acid, arabinofuranose cleavage
10BWT4_2165	10BWT4	2165	Terracidiphlus	GH2(42-613)	GH2	Y(1-32)	galactose, mannose, glucuronic acid, arabinofuranose cleavage
0BWT1_100	0BWT1	100	Terracidiphlus	GH2(45-541)	GH2	N	galactose, mannose, glucuronic acid, arabinofuranose cleavage
0BWT2_2985	0BWT2	2985	Terracidiphlus	GH2(49-545)	GH2	Y(1-38)	galactose, mannose, glucuronic acid, arabinofuranose cleavage
0BWT2_2379	0BWT2	2379	Terracidiphlus	GH2(51-726)	GH2	Y(1-24)	galactose, mannose, glucuronic acid, arabinofuranose cleavage
0BWT1_1600	0BWT1	1600	Terracidiphlus	GH2(51-743)	GH2	Y(1-26)	galactose, mannose, glucuronic acid, arabinofuranose cleavage
0UNS5_780	0UNS5	780	Terracidiphlus	GH2(52-618)+CBM	GH2	Y(1-27)	galactose, mannose, glucuronic acid, arabinofuranose cleavage
0UNS4_800	0UNS4	800	Terracidiphlus	GH2(52-726)	GH2	Y(1-29)	galactose, mannose, glucuronic acid, arabinofuranose cleavage
10BWT4_1646	10BWT4	1646	Terracidiphlus	GH2(57-614)	GH2	Y(1-29)	galactose, mannose, glucuronic acid, arabinofuranose cleavage
0BWT2_3087	0BWT2	3087	Terracidiphlus	GH2(58-705)	GH2	N	galactose, mannose, glucuronic acid, arabinofuranose cleavage
0BWT2_884	0BWT2	884	Terracidiphlus	GH2(58-770)+CBM	GH2	N	galactose, mannose, glucuronic acid, arabinofuranose cleavage
0BWT1_2051	0BWT1	2051	Terracidiphlus	GH2(62-491)	GH2	Y(1-27)	galactose, mannose, glucuronic acid, arabinofuranose cleavage
0BWT2_137	0BWT2	137	Terracidiphlus	GH2(68-519)	GH2	N	galactose, mannose, glucuronic acid, arabinofuranose cleavage
0BWT2_1614	0BWT2	1614	Terracidiphlus	GH2(70-598)	GH2	N	galactose, mannose, glucuronic acid, arabinofuranose cleavage
0UNS4_2699	0UNS4	2699	Terracidiphlus	GH2(8-733)+CBM3	CBM32+GH2+(	N	galactose, mannose, glucuronic acid, arabinofuranose cleavage
0BWT2_643	0BWT2	643	Terracidiphlus	GH2(84-667)	GH2	Y(1-30)	galactose, mannose, glucuronic acid, arabinofuranose cleavage
10BWT3_3782	10BWT3	3782	Holophaga	GH13_26(1-344)	GH13_26+GH7	N	Glucan
0BWT1_473	0BWT1	473	Terracidiphlus	GH144(320-741)	GH144	Y(1-33)	Glucan
0UNS5_78	0UNS5	78	Terracidiphlus	GH144(38-424)	GH144	Y(1-39)	Glucan
0UNS4_1504	0UNS4	1504	Terracidiphlus	GH144(40-428)	GH144	Y(1-33)	Glucan
10BWT4_1574	10BWT4	1574	Terracidiphlus	GH144(43-431)	GH144	N	Glucan
0BWT1_471	0BWT1	471	Terracidiphlus	GH144(44-432)	GH144	Y(1-36)	Glucan
0BWT1_470	0BWT1	470	Terracidiphlus	GH144(60-467)	GH144	Y(1-23)	Glucan
0BWT1_970	0BWT1	970	Terracidiphlus	GH149(20-1139)	GH149	N	glucan
0UNS4_2721	0UNS4	2721	Terracidiphlus	GH6(63-440)+CBM	CBM3+GH6	Y(1-39)	glucan
0UNS5_1703	0UNS5	1703	Terracidiphlus	GH6(63-440)+CBM	CBM3+GH6	Y(1-39)	glucan
0UNS4_1805	0UNS4	1805	Terracidiphlus	GH77(1-486)	GH77	N	glucan
10UNS1_3279	10UNS1	3279	Holophaga	GH77(1-510)+GH1	GH13_26+GH7	N	glucan
0BWT2_244	0BWT2	244	Terracidiphlus	GH77(11-480)	GH77	N	glucan
0BWT1_1407	0BWT1	1407	Terracidiphlus	GH77(11-495)	GH77	N	glucan
0UNS5_591	0UNS5	591	Terracidiphlus	GH77(11-495)	GH77	N	glucan
5AWT5_874	5AWT5	874	Holophaga	GH77(189-720)+GI	GH13_26+GH7	N	glucan
10BWT3_2805	10BWT3	2805	Holophaga	GH77(213-760)	GH13_26+GH7	N	glucan
10AWT2_1556	10AWT2	1556	Holophaga	GH77(213-760)+GI	GH13_26+GH7	N	glucan
5AWT2_3794	5AWT2	3794	Holophaga	GH77(213-760)+GI	GH13_26+GH7	N	glucan
5BWT7_2072	5BWT7	2072	Holophaga	GH77(213-760)+GI	GH13_26+GH7	N	glucan
5UNS4_1486	5UNS4	1486	Holophaga	GH77(213-760)+GI	GH13_26+GH7	N	glucan
0BWT1_957	0BWT1	957	Terracidiphlus	GH9(129-712)	GH9	Y(1-29)	glucan
0BWT2_1330	0BWT2	1330	Terracidiphlus	GH9(132-583)	GH9	N	glucan
0BWT2_1881	0BWT2	1881	Terracidiphlus	GH9(132-583)	GH9	Y(1-38)	glucan
0BWT1_2600	0BWT1	2600	Terracidiphlus	GH9(134-582)	GH9	N	glucan
0BWT2_2613	0BWT2	2613	Terracidiphlus	GH55(155-617)	GH55	Y(1-34)	glucan (laminarin)
0BWT2_3692	0BWT2	3692	Terracidiphlus	GH55(165-429)	GH55	Y(1-24)	glucan (laminarin)
10BWT4_1907	10BWT4	1907	Terracidiphlus	GH55(269-526)	GH55	Y(1-26)	glucan (laminarin)
0BWT2_1353	0BWT2	1353	Terracidiphlus	GH55(277-558)	GH55	N	glucan (laminarin)
10BWT4_12	10BWT4	12	Terracidiphlus	GH1(36-476)	GH1	Y(1-31)	Glucose and galactose (also mannose and fucose)
0UNS4_1705	0UNS4	1705	Terracidiphlus	GH1(43-484)	GH1	Y(1-35)	Glucose and galactose (also mannose and fucose)
0UNS5_355	0UNS5	355	Terracidiphlus	GH1(43-484)	GH1	Y(1-33)	Glucose and galactose (also mannose and fucose)
10BWT3_3284	10BWT3	3284	Holophaga	GH1(7-404)	GH1	N	Glucose and galactose (also mannose and fucose)
10AWT2_182	10AWT2	182	Holophaga	GH1(7-450)	GH1	N	Glucose and galactose (also mannose and fucose)
10UNS1_895	10UNS1	895	Holophaga	GH1(7-450)	GH1	N	Glucose and galactose (also mannose and fucose)
5AWT2_776	5AWT2	776	Holophaga	GH1(7-450)	GH1	N	Glucose and galactose (also mannose and fucose)
5BWT7_2697	5BWT7	2697	Holophaga	GH1(7-450)	GH1	N	Glucose and galactose (also mannose and fucose)
5UNS4_488	5UNS4	488	Holophaga	GH1(7-450)	GH1	N	Glucose and galactose (also mannose and fucose)
10BWT4_1736	10BWT4	1736	Terracidiphlus	GH97(21-401)	GH97	N	glucose, galactose cleavage
10BWT4_3203	10BWT4	3203	Terracidiphlus	GH97(29-661)	GH97	Y(1-30)	glucose, galactose cleavage
10BWT4_3043	10BWT4	3043	Terracidiphlus	PL38(17-300)	PL38	N	glucuronan
0BWT2_135	0BWT2	135	Terracidiphlus	PL38(68-339)	PL38	Y(1-38)	glucuronan
0UNS4_2247	0UNS4	2247	Terracidiphlus	CBM57(186-330)+	CBM57	N	Glycan
0BWT2_1656	0BWT2	1656	Terracidiphlus	CBM57(208-354)+	CBM57	N	Glycan
10BWT4_106	10BWT4	106	Terracidiphlus	CBM57(209-344)+	CBM57	N	Glycan
0BWT1_2255	0BWT1	2255	Terracidiphlus	CBM57(221-355)+	CBM57	N	Glycan
0BWT1_958	0BWT1	958	Terracidiphlus	GH20(163-480)	GH20	N	glycans, glycoproteins, glycolipids,
0UNS4_1350	0UNS4	1350	Terracidiphlus	GH20(167-461)	GH20	Y(1-64)	glycans, glycoproteins, glycolipids,
0BWT1_3121	0BWT1	3121	Terracidiphlus	GH20(168-464)	GH20	Y(1-26)	glycans, glycoproteins, glycolipids,

0UNS5_1471	0UNS5	1471	Terracidiphlus	GH20(169-463)	GH20	N	glycans, glycoproteins, glycolipids,
0BWT2_3062	0BWT2	3062	Terracidiphlus	GH20(174-469)	GH20	Y(1-22)	glycans, glycoproteins, glycolipids,
0BWT2_346	0BWT2	346	Terracidiphlus	GH20(190-487)	GH20	Y(1-38)	glycans, glycoproteins, glycolipids,
10BWT4_2014	10BWT4	2014	Terracidiphlus	GH20(202-499)	GH20	N	glycans, glycoproteins, glycolipids,
10BWT3_4185	10BWT3	4185	Holophaga	GH13_9(1-234)	CBM48+GH13_	N	glycogen synthesis
10AWT2_2898	10AWT2	2898	Holophaga	GH13_9(1-238)	CBM48+GH13_	N	glycogen synthesis
0UNS5_839	0UNS5	839	Terracidiphlus	GH79(48-352)	GH79	Y(1-25)	glycosaminoglycan, Hyaluronan, heparan sulfate
0UNS4_1371	0UNS4	1371	Terracidiphlus	GH79(49-353)	GH79	Y(1-25)	glycosaminoglycan, Hyaluronan, heparan sulfate
10BWT4_1292	10BWT4	1292	Terracidiphlus	GH79(50-348)	GH79	Y(1-27)	glycosaminoglycan, Hyaluronan, heparan sulfate
10BWT4_2318	10BWT4	2318	Terracidiphlus	GH79(96-522)	GH79	Y(1-27)	glycosaminoglycan, Hyaluronan, heparan sulfate
0UNS4_1969	0UNS4	1969	Terracidiphlus	GH105(53-392)	GH105	Y(1-24)	Glycosaminoglycans
10BWT4_3312	10BWT4	3312	Terracidiphlus	GH105(53-395)	GH105	Y(1-23)	Glycosaminoglycans
0BWT1_706	0BWT1	706	Terracidiphlus	GH105(54-393)	GH105	Y(1-24)	Glycosaminoglycans
0UNS4_2816	0UNS4	2816	Terracidiphlus	GH105(55-361)	GH105	Y(1-26)	Glycosaminoglycans
0BWT2_488	0BWT2	488	Terracidiphlus	GH105(55-393)	GH105	Y(1-24)	Glycosaminoglycans
0BWT2_2143	0BWT2	2143	Terracidiphlus	GH105(55-395)	GH105	Y(1-28)	Glycosaminoglycans
0UNS5_101	0UNS5	101	Terracidiphlus	GH105(56-396)	GH105	Y(1-26)	Glycosaminoglycans
0BWT1_2191	0BWT1	2191	Terracidiphlus	GH105(57-363)	GH105	Y(1-27)	Glycosaminoglycans
10BWT4_3280	10BWT4	3280	Terracidiphlus	GH105(62-361)	GH105	Y(1-23)	Glycosaminoglycans
0BWT2_714	0BWT2	714	Terracidiphlus	GH105(78-381)	GH105	Y(1-32)	Glycosaminoglycans
0BWT2_469	0BWT2	469	Terracidiphlus	GH105(85-394)	GH105	Y(1-28)	Glycosaminoglycans
0BWT2_133	0BWT2	133	Terracidiphlus	GH105(90-394)	GH105	Y(1-41)	Glycosaminoglycans
0UNS5_561	0UNS5	561	Terracidiphlus	GH154(1-251)	GH154	N	glycosaminoglycans
0BWT2_274	0BWT2	274	Terracidiphlus	GH154(16-374)	GH154	N	glycosaminoglycans
0UNS4_801	0UNS4	801	Terracidiphlus	GH154(31-381)	GH154	N	glycosaminoglycans
0UNS5_949	0UNS5	949	Terracidiphlus	GT1(12-422)	GT1	N	Glycosyl transferase
0BWT2_1874	0BWT2	1874	Terracidiphlus	GT1(14-420)	GT1	N	Glycosyl transferase
0UNS4_2686	0UNS4	2686	Terracidiphlus	GT1(14-421)	GT1	N	Glycosyl transferase
0BWT1_3283	0BWT1	3283	Terracidiphlus	GT1(19-425)	GT1	N	Glycosyl transferase
10BWT4_1504	10BWT4	1504	Terracidiphlus	GT1(69-421)	GT1	N	Glycosyl transferase
0BWT2_1754	0BWT2	1754	Terracidiphlus	GT19(4-363)	GT19	N	Glycosyl transferase
0UNS4_262	0UNS4	262	Terracidiphlus	GT19(5-365)	GT19	N	Glycosyl transferase
0UNS5_1503	0UNS5	1503	Terracidiphlus	GT19(5-367)	GT19	N	Glycosyl transferase
0BWT1_148	0BWT1	148	Terracidiphlus	GT19(5-370)	GT19	N	Glycosyl transferase
0UNS4_2326	0UNS4	2326	Terracidiphlus	GT2_Glyco_tranf_2	GT2	Y(1-24)	Glycosyl transferase
0UNS5_639	0UNS5	639	Terracidiphlus	GT2_Glyco_tranf_2	GT2	N	Glycosyl transferase
0BWT1_2281	0BWT1	2281	Terracidiphlus	GT2_Glyco_tranf_2	GT2	N	Glycosyl transferase
10BWT4_202	10BWT4	202	Terracidiphlus	GT2_Glyco_tranf_2	GT2	N	Glycosyl transferase
10AWT2_1961	10AWT2	1961	Holophaga	GT2_Glyco_tranf_2	GT2	N	Glycosyl transferase
10BWT3_1012	10BWT3	1012	Holophaga	GT2_Glyco_tranf_2	GT2	N	Glycosyl transferase
10UNS1_1322	10UNS1	1322	Holophaga	GT2_Glyco_tranf_2	GT2	N	Glycosyl transferase
5AWT2_1981	5AWT2	1981	Holophaga	GT2_Glyco_tranf_2	GT2	N	Glycosyl transferase
5AWT5_1779	5AWT5	1779	Holophaga	GT2_Glyco_tranf_2	GT2	N	Glycosyl transferase
5BWT7_1431	5BWT7	1431	Holophaga	GT2_Glyco_tranf_2	GT2	N	Glycosyl transferase
5UNS4_2403	5UNS4	2403	Holophaga	GT2_Glyco_tranf_2	GT2	N	Glycosyl transferase
0BWT2_591	0BWT2	591	Terracidiphlus	GT2_Glycos_transf	GT2	N	Glycosyl transferase
0BWT2_38	0BWT2	38	Terracidiphlus	GT2_Glycos_transf	GT2	N	Glycosyl transferase
0UNS5_1713	0UNS5	1713	Terracidiphlus	GT2_Glycos_transf	GT2	N	Glycosyl transferase
10BWT4_911	10BWT4	911	Terracidiphlus	GT2_Glycos_transf	GT2	N	Glycosyl transferase
0UNS4_797	0UNS4	797	Terracidiphlus	GT2_Glycos_transf	GT2	N	Glycosyl transferase
0BWT2_616	0BWT2	616	Terracidiphlus	GT2_Glycos_transf	GT2	N	Glycosyl transferase
0UNS5_1349	0UNS5	1349	Terracidiphlus	GT2_Glycos_transf	GT2	N	Glycosyl transferase
0BWT2_1961	0BWT2	1961	Terracidiphlus	GT2_Glycos_transf	GT2	N	Glycosyl transferase
0BWT1_3221	0BWT1	3221	Terracidiphlus	GT2_Glycos_transf	GT2	N	Glycosyl transferase
0BWT1_2355	0BWT1	2355	Terracidiphlus	GT2_Glycos_transf	GT2	N	Glycosyl transferase
5BWT7_4825	5BWT7	4825	Holophaga	GT2_Glycos_transf	GT2	N	Glycosyl transferase
0UNS4_500	0UNS4	500	Terracidiphlus	GT2_Glycos_transf	GT2	N	Glycosyl transferase
0UNS5_2323	0UNS5	2323	Terracidiphlus	GT2_Glycos_transf	GT2	N	Glycosyl transferase
0BWT1_1923	0BWT1	1923	Terracidiphlus	GT2_Glycos_transf	GT2	N	Glycosyl transferase
5AWT5_915	5AWT5	915	Holophaga	GT2_Glycos_transf	GT2	N	Glycosyl transferase
10AWT2_4601	10AWT2	4601	Holophaga	GT2_Glycos_transf	GT2	N	Glycosyl transferase
10BWT3_2428	10BWT3	2428	Holophaga	GT2_Glycos_transf	GT2	N	Glycosyl transferase
10UNS1_780	10UNS1	780	Holophaga	GT2_Glycos_transf	GT2	N	Glycosyl transferase
5AWT2_713	5AWT2	713	Holophaga	GT2_Glycos_transf	GT2	N	Glycosyl transferase
5UNS4_1803	5UNS4	1803	Holophaga	GT2_Glycos_transf	GT2	N	Glycosyl transferase
0BWT2_3918	0BWT2	3918	Terracidiphlus	GT2_Glycos_transf	GT2	N	Glycosyl transferase
0UNS4_592	0UNS4	592	Terracidiphlus	GT2_Glycos_transf	GT2	N	Glycosyl transferase
10BWT4_1364	10BWT4	1364	Terracidiphlus	GT2_Glycos_transf	GT2	N	Glycosyl transferase

5UNS4_1617	5UNS4	1617	Holophaga	GT2_Glycos_transf	GT2	N	Glycosyl transferase
10UNS1_264	10UNS1	264	Holophaga	GT2_Glycos_transf	GT2	N	Glycosyl transferase
0BWT1_2498	0BWT1	2498	Terracidiphlus	GT2_Glycos_transf	GT2	N	Glycosyl transferase
0BWT2_1290	0BWT2	1290	Terracidiphlus	GT2_Glycos_transf	GT2	N	Glycosyl transferase
0UNS5_1222	0UNS5	1222	Terracidiphlus	GT2_Glycos_transf	GT2	N	Glycosyl transferase
0UNS4_1915	0UNS4	1915	Terracidiphlus	GT2_Glycos_transf	GT2	N	Glycosyl transferase
5AWT5_2349	5AWT5	2349	Holophaga	GT2_Glycos_transf	GT2	N	Glycosyl transferase
5AWT5_1665	5AWT5	1665	Holophaga	GT2_Glycos_transf	GT2	N	Glycosyl transferase
10AWT2_3670	10AWT2	3670	Holophaga	GT2_Glycos_transf	GT2	N	Glycosyl transferase
10BWT3_2257	10BWT3	2257	Holophaga	GT2_Glycos_transf	GT2	N	Glycosyl transferase
10UNS1_2277	10UNS1	2277	Holophaga	GT2_Glycos_transf	GT2	N	Glycosyl transferase
5AWT2_3783	5AWT2	3783	Holophaga	GT2_Glycos_transf	GT2	N	Glycosyl transferase
5UNS4_679	5UNS4	679	Holophaga	GT2_Glycos_transf	GT2	N	Glycosyl transferase
10BWT4_2151	10BWT4	2151	Terracidiphilus	GT20(251-717)	GT20	Y(1-48)	Glycosyl transferase
0BWT1_824	0BWT1	824	Terracidiphlus	GT20(254-721)	GT20	N	Glycosyl transferase
0UNS4_167	0UNS4	167	Terracidiphlus	GT20(273-753)	GT20	N	Glycosyl transferase
10BWT4_2022	10BWT4	2022	Terracidiphilus	GT21(1-228)	GT21	N	Glycosyl transferase
0UNS4_2250	0UNS4	2250	Terracidiphlus	GT21(47-283)	GT21	N	Glycosyl transferase
0UNS5_213	0UNS5	213	Terracidiphlus	GT21(47-283)	GT21	N	Glycosyl transferase
0BWT1_106	0BWT1	106	Terracidiphlus	GT21(47-286)	GT21	N	Glycosyl transferase
0BWT2_209	0BWT2	209	Terracidiphlus	GT21(47-288)	GT21	N	Glycosyl transferase
0UNS4_1557	0UNS4	1557	Terracidiphlus	GT21(49-279)	GT21	N	Glycosyl transferase
0UNS5_344	0UNS5	344	Terracidiphlus	GT21(49-279)	GT21	N	Glycosyl transferase
0BWT1_655	0BWT1	655	Terracidiphlus	GT22(22-368)	GT22	N	Glycosyl transferase
0BWT1_1042	0BWT1	1042	Terracidiphlus	GT28(181-346)	GT28	N	Glycosyl transferase
0BWT2_564	0BWT2	564	Terracidiphlus	GT28(181-346)	GT28	N	Glycosyl transferase
0UNS4_2877	0UNS4	2877	Terracidiphlus	GT28(182-347)	GT28	N	Glycosyl transferase
0UNS5_1025	0UNS5	1025	Terracidiphlus	GT28(182-347)	GT28	N	Glycosyl transferase
10BWT4_1126	10BWT4	1126	Terracidiphilus	GT28(185-349)	GT28	N	Glycosyl transferase
0UNS4_2637	0UNS4	2637	Terracidiphlus	GT30(35-213)	GT30	N	Glycosyl transferase
0UNS5_287	0UNS5	287	Terracidiphlus	GT30(35-213)	GT30	N	Glycosyl transferase
10BWT4_2668	10BWT4	2668	Terracidiphilus	GT30(35-217)	GT30	N	Glycosyl transferase
0BWT2_27	0BWT2	27	Terracidiphlus	GT30(36-215)	GT30	N	Glycosyl transferase
0BWT1_1955	0BWT1	1955	Terracidiphlus	GT30(43-219)	GT30	N	Glycosyl transferase
5BWT7_1670	5BWT7	1670	Holophaga	GT35(1-264)	GT35	N	Glycosyl transferase
5AWT5_2564	5AWT5	2564	Holophaga	GT35(101-804)	GT35	N	Glycosyl transferase
0BWT2_1158	0BWT2	1158	Terracidiphlus	GT35(102-814)	GT35	N	Glycosyl transferase
10AWT2_2208	10AWT2	2208	Holophaga	GT35(105-811)	GT35	N	Glycosyl transferase
10BWT3_1465	10BWT3	1465	Holophaga	GT35(105-811)	GT35	N	Glycosyl transferase
5BWT7_2131	5BWT7	2131	Holophaga	GT35(105-811)	GT35	N	Glycosyl transferase
5UNS4_1547	5UNS4	1547	Holophaga	GT35(105-811)	GT35	N	Glycosyl transferase
10BWT4_746	10BWT4	746	Terracidiphilus	GT35(111-823)	GT35	N	Glycosyl transferase
5BWT7_4775	5BWT7	4775	Holophaga	GT35(143-501)	GT35	N	Glycosyl transferase
0BWT1_1128	0BWT1	1128	Terracidiphlus	GT35(180-511)	GT35	N	Glycosyl transferase
10BWT4_1511	10BWT4	1511	Terracidiphilus	GT35(180-517)	GT35	N	Glycosyl transferase
0BWT2_156	0BWT2	156	Terracidiphlus	GT35(180-522)	GT35	N	Glycosyl transferase
0UNS5_1817	0UNS5	1817	Terracidiphlus	GT35(180-522)	GT35	N	Glycosyl transferase
0UNS4_2600	0UNS4	2600	Terracidiphlus	GT35(180-524)	GT35	N	Glycosyl transferase
10BWT3_3184	10BWT3	3184	Holophaga	GT35(227-589)	GT35	N	Glycosyl transferase
10UNS1_4415	10UNS1	4415	Holophaga	GT35(247-631)	GT35	N	Glycosyl transferase
5AWT2_2891	5AWT2	2891	Holophaga	GT35(247-631)	GT35	N	Glycosyl transferase
5UNS4_3622	5UNS4	3622	Holophaga	GT35(247-631)	GT35	N	Glycosyl transferase
5AWT5_3086	5AWT5	3086	Holophaga	GT35(259-608)	GT35	N	Glycosyl transferase
10BWT3_2702	10BWT3	2702	Holophaga	GT35(259-617)	GT35	N	Glycosyl transferase
10UNS1_3872	10UNS1	3872	Holophaga	GT35(259-617)	GT35	N	Glycosyl transferase
5UNS4_2639	5UNS4	2639	Holophaga	GT35(259-617)	GT35	N	Glycosyl transferase
10AWT2_1908	10AWT2	1908	Holophaga	GT35(259-630)	GT35	N	Glycosyl transferase
5AWT2_299	5AWT2	299	Holophaga	GT35(259-630)	GT35	N	Glycosyl transferase
10AWT2_3984	10AWT2	3984	Holophaga	GT35(26-409)	GT35	N	Glycosyl transferase
0BWT1_3213	0BWT1	3213	Terracidiphlus	GT35(265-630)	GT35	N	Glycosyl transferase
10AWT2_4448	10AWT2	4448	Holophaga	GT35(266-627)	GT35	N	Glycosyl transferase
5AWT2_3665	5AWT2	3665	Holophaga	GT35(266-628)	GT35	N	Glycosyl transferase
5UNS4_380	5UNS4	380	Holophaga	GT35(266-629)	GT35	N	Glycosyl transferase
10BWT4_712	10BWT4	712	Terracidiphilus	GT35(267-626)	GT35	N	Glycosyl transferase
0BWT1_2650	0BWT1	2650	Terracidiphlus	GT35(268-623)	GT35	N	Glycosyl transferase
0BWT2_1541	0BWT2	1541	Terracidiphlus	GT35(269-621)	GT35	N	Glycosyl transferase
0BWT1_487	0BWT1	487	Terracidiphlus	GT35(274-635)	GT35	N	Glycosyl transferase
0BWT1_3552	0BWT1	3552	Terracidiphlus	GT35(275-652)	GT35	N	Glycosyl transferase

10BWT4_654	10BWT4	654	Terracidiphilus	GT35(54-414)	GT35	N	Glycosyl transferase
10BWT3_4679	10BWT3	4679	Holophaga	GT35(61-445)	GT35	N	Glycosyl transferase
10UNS1_1526	10UNS1	1526	Holophaga	GT35(74-780)	GT35	N	Glycosyl transferase
10AWT2_3705	10AWT2	3705	Holophaga	GT4(159-291)	GT4	N	Glycosyl transferase
10UNS1_1259	10UNS1	1259	Holophaga	GT4(159-291)	GT4	N	Glycosyl transferase
5AWT2_3131	5AWT2	3131	Holophaga	GT4(159-291)	GT4	N	Glycosyl transferase
5BWT7_3574	5BWT7	3574	Holophaga	GT4(159-291)	GT4	N	Glycosyl transferase
5UNS4_1490	5UNS4	1490	Holophaga	GT4(159-291)	GT4	N	Glycosyl transferase
10AWT2_4349	10AWT2	4349	Holophaga	GT4(192-339)	GT4	N	Glycosyl transferase
10BWT3_3937	10BWT3	3937	Holophaga	GT4(192-339)	GT4	N	Glycosyl transferase
10UNS1_3033	10UNS1	3033	Holophaga	GT4(192-339)	GT4	N	Glycosyl transferase
5AWT2_1391	5AWT2	1391	Holophaga	GT4(192-339)	GT4	N	Glycosyl transferase
5BWT7_622	5BWT7	622	Holophaga	GT4(192-339)	GT4	N	Glycosyl transferase
0BWT2_3250	0BWT2	3250	Terracidiphlus	GT4(195-348)	GT4	N	Glycosyl transferase
0UNS4_518	0UNS4	518	Terracidiphlus	GT4(199-348)	GT4	N	Glycosyl transferase
0UNS5_2216	0UNS5	2216	Terracidiphlus	GT4(200-342)	GT4	N	Glycosyl transferase
0BWT1_757	0BWT1	757	Terracidiphlus	GT4(200-348)	GT4	N	Glycosyl transferase
10AWT2_1241	10AWT2	1241	Holophaga	GT4(200-359)	GT4	N	Glycosyl transferase
5AWT2_854	5AWT2	854	Holophaga	GT4(200-359)	GT4	N	Glycosyl transferase
5BWT7_764	5BWT7	764	Holophaga	GT4(200-359)	GT4	N	Glycosyl transferase
10BWT4_293	10BWT4	293	Terracidiphilus	GT4(202-344)	GT4	N	Glycosyl transferase
10BWT4_1376	10BWT4	1376	Terracidiphilus	GT4(204-355)	GT4	N	Glycosyl transferase
0BWT2_1017	0BWT2	1017	Terracidiphlus	GT4(204-358)	GT4	N	Glycosyl transferase
0UNS5_2073	0UNS5	2073	Terracidiphlus	GT4(205-349)	GT4	N	Glycosyl transferase
0BWT1_1891	0BWT1	1891	Terracidiphlus	GT4(207-352)	GT4	N	Glycosyl transferase
0UNS4_1147	0UNS4	1147	Terracidiphlus	GT4(215-383)	GT4	N	Glycosyl transferase
0BWT2_1213	0BWT2	1213	Terracidiphlus	GT4(217-351)	GT4	N	Glycosyl transferase
10BWT4_496	10BWT4	496	Terracidiphilus	GT4(218-383)	GT4	N	Glycosyl transferase
0BWT1_2691	0BWT1	2691	Terracidiphlus	GT4(218-383)	GT4	N	Glycosyl transferase
0BWT2_910	0BWT2	910	Terracidiphlus	GT4(218-383)	GT4	N	Glycosyl transferase
0UNS5_142	0UNS5	142	Terracidiphlus	GT4(218-383)	GT4	N	Glycosyl transferase
10BWT4_362	10BWT4	362	Terracidiphilus	GT4(219-357)	GT4	N	Glycosyl transferase
0UNS4_623	0UNS4	623	Terracidiphlus	GT4(220-379)	GT4	N	Glycosyl transferase
0UNS5_1879	0UNS5	1879	Terracidiphlus	GT4(221-353)	GT4	N	Glycosyl transferase
0BWT2_374	0BWT2	374	Terracidiphlus	GT4(226-388)	GT4	N	Glycosyl transferase
10AWT2_2964	10AWT2	2964	Holophaga	GT4(228-387)	GT4	N	Glycosyl transferase
10BWT3_603	10BWT3	603	Holophaga	GT4(228-387)	GT4	N	Glycosyl transferase
5AWT2_474	5AWT2	474	Holophaga	GT4(228-387)	GT4	N	Glycosyl transferase
5BWT7_3859	5BWT7	3859	Holophaga	GT4(228-387)	GT4	N	Glycosyl transferase
5UNS4_3568	5UNS4	3568	Holophaga	GT4(228-387)	GT4	N	Glycosyl transferase
0UNS4_2930	0UNS4	2930	Terracidiphlus	GT4(236-380)	GT4	N	Glycosyl transferase
0UNS5_679	0UNS5	679	Terracidiphlus	GT4(237-380)	GT4	N	Glycosyl transferase
0BWT1_2990	0BWT1	2990	Terracidiphlus	GT4(241-389)	GT4	N	Glycosyl transferase
10AWT2_680	10AWT2	680	Holophaga	GT4(250-419)	GT4	N	Glycosyl transferase
10BWT3_1320	10BWT3	1320	Holophaga	GT4(250-419)	GT4	N	Glycosyl transferase
5AWT2_2573	5AWT2	2573	Holophaga	GT4(250-419)	GT4	N	Glycosyl transferase
5BWT7_4712	5BWT7	4712	Holophaga	GT4(250-419)	GT4	N	Glycosyl transferase
5AWT5_2704	5AWT5	2704	Holophaga	GT4(251-417)	GT4	N	Glycosyl transferase
10UNS1_1495	10UNS1	1495	Holophaga	GT4(252-421)	GT4	N	Glycosyl transferase
5BWT7_3256	5BWT7	3256	Holophaga	GT4(329-471)	GT4	N	Glycosyl transferase
10AWT2_1211	10AWT2	1211	Holophaga	GT4(729-871)	GT4	N	Glycosyl transferase
10BWT3_1780	10BWT3	1780	Holophaga	GT4(729-871)	GT4	N	Glycosyl transferase
10UNS1_2813	10UNS1	2813	Holophaga	GT4(729-871)	GT4	N	Glycosyl transferase
5AWT2_280	5AWT2	280	Holophaga	GT4(729-871)	GT4	N	Glycosyl transferase
5UNS4_3792	5UNS4	3792	Holophaga	GT4(729-871)	GT4	N	Glycosyl transferase
0UNS4_756	0UNS4	756	Terracidiphlus	GT41(22-606)	GT41	N	Glycosyl transferase
0UNS5_1524	0UNS5	1524	Terracidiphlus	GT41(22-607)	GT41	N	Glycosyl transferase
5AWT5_749	5AWT5	749	Holophaga	GT41(270-750)	GT41	N	Glycosyl transferase
5UNS4_423	5UNS4	423	Holophaga	GT41(275-760)	GT41	N	Glycosyl transferase
10AWT2_2821	10AWT2	2821	Holophaga	GT41(277-760)	GT41	N	Glycosyl transferase
10BWT3_2716	10BWT3	2716	Holophaga	GT41(277-760)	GT41	N	Glycosyl transferase
10UNS1_2734	10UNS1	2734	Holophaga	GT41(277-760)	GT41	N	Glycosyl transferase
5AWT2_29	5AWT2	29	Holophaga	GT41(277-760)	GT41	N	Glycosyl transferase
5BWT7_1200	5BWT7	1200	Holophaga	GT41(277-760)	GT41	N	Glycosyl transferase
10UNS1_3723	10UNS1	3723	Holophaga	GT5(1-464)	GT5	N	Glycosyl transferase
0BWT2_3117	0BWT2	3117	Terracidiphlus	GT5(2-396)	GT5	N	Glycosyl transferase
10AWT2_1909	10AWT2	1909	Holophaga	GT5(2-473)	GT5	N	Glycosyl transferase
5AWT2_298	5AWT2	298	Holophaga	GT5(2-473)	GT5	N	Glycosyl transferase

5AWT5_1413	5AWT5	1413	Holophaga	GT5(2-473)	GT5	N	Glycosyl transferase
5BWT7_1443	5BWT7	1443	Holophaga	GT5(2-473)	GT5	N	Glycosyl transferase
5UNS4_3385	5UNS4	3385	Holophaga	GT5(2-473)	GT5	N	Glycosyl transferase
0UNS4_409	0UNS4	409	Terracidiphlus	GT5(2-477)	GT5	N	Glycosyl transferase
10BWT4_2740	10BWT4	2740	Terracidiphlus	GT5(2-478)	GT5	N	Glycosyl transferase
0BWT1_1688	0BWT1	1688	Terracidiphlus	GT5(2-478)	GT5	N	Glycosyl transferase
0UNS5_2467	0UNS5	2467	Terracidiphlus	GT5(2-478)	GT5	N	Glycosyl transferase
5AWT5_108	5AWT5	108	Holophaga	GT5(2-523)	GT5	N	Glycosyl transferase
10AWT2_4147	10AWT2	4147	Holophaga	GT51(1-85)	GT51	N	Glycosyl transferase
0UNS4_1263	0UNS4	1263	Terracidiphlus	GT51(103-278)	GT51	N	Glycosyl transferase
10BWT4_704	10BWT4	704	Terracidiphlus	GT51(104-279)	GT51	N	Glycosyl transferase
0UNS5_1858	0UNS5	1858	Terracidiphlus	GT51(110-285)	GT51	N	Glycosyl transferase
0UNS4_1598	0UNS4	1598	Terracidiphlus	GT51(164-337)	GT51	N	Glycosyl transferase
10BWT4_1211	10BWT4	1211	Terracidiphlus	GT51(166-339)	GT51	N	Glycosyl transferase
0UNS5_625	0UNS5	625	Terracidiphlus	GT51(166-339)	GT51	N	Glycosyl transferase
0BWT1_1676	0BWT1	1676	Terracidiphlus	GT51(168-339)	GT51	N	Glycosyl transferase
5AWT5_2401	5AWT5	2401	Holophaga	GT51(33-207)	GT51	N	Glycosyl transferase
10UNS1_1540	10UNS1	1540	Holophaga	GT51(34-209)	GT51	N	Glycosyl transferase
5UNS4_4609	5UNS4	4609	Holophaga	GT51(34-209)	GT51	N	Glycosyl transferase
10BWT3_4507	10BWT3	4507	Holophaga	GT51(38-213)	GT51	N	Glycosyl transferase
5AWT2_2862	5AWT2	2862	Holophaga	GT51(38-213)	GT51	N	Glycosyl transferase
5BWT7_1479	5BWT7	1479	Holophaga	GT51(38-213)	GT51	N	Glycosyl transferase
5AWT5_1338	5AWT5	1338	Holophaga	GT51(70-250)	GT51	N	Glycosyl transferase
10AWT2_2382	10AWT2	2382	Holophaga	GT51(71-251)	GT51	N	Glycosyl transferase
10BWT3_2565	10BWT3	2565	Holophaga	GT51(71-251)	GT51	N	Glycosyl transferase
5AWT2_3421	5AWT2	3421	Holophaga	GT51(71-251)	GT51	N	Glycosyl transferase
5BWT7_1405	5BWT7	1405	Holophaga	GT51(71-251)	GT51	N	Glycosyl transferase
0BWT1_2370	0BWT1	2370	Terracidiphlus	GT51(85-260)	GT51	N	Glycosyl transferase
0BWT2_1494	0BWT2	1494	Terracidiphlus	GT51(85-260)	GT51	N	Glycosyl transferase
0BWT2_3552	0BWT2	3552	Terracidiphlus	GT51(98-270)	GT51	N	Glycosyl transferase
0BWT1_399	0BWT1	399	Terracidiphlus	GT83(13-572)	GT83	N	Glycosyl transferase
0UNS4_63	0UNS4	63	Terracidiphlus	GT83(18-556)	GT83	N	Glycosyl transferase
0UNS5_617	0UNS5	617	Terracidiphlus	GT83(18-574)	GT83	N	Glycosyl transferase
0UNS4_2186	0UNS4	2186	Terracidiphlus	GT83(29-523)	GT83	N	Glycosyl transferase
0UNS5_875	0UNS5	875	Terracidiphlus	GT83(30-474)	GT83	N	Glycosyl transferase
0BWT2_1979	0BWT2	1979	Terracidiphlus	GT83(36-576)	GT83	N	Glycosyl transferase
0BWT1_2180	0BWT1	2180	Terracidiphlus	GT83(60-506)	GT83	N	Glycosyl transferase
0BWT2_3175	0BWT2	3175	Terracidiphlus	GT83(69-625)	GT83	N	Glycosyl transferase
0BWT2_440	0BWT2	440	Terracidiphlus	GT83(74-528)	GT83	N	Glycosyl transferase
10BWT4_1429	10BWT4	1429	Terracidiphlus	GT83(8-496)	GT83	N	Glycosyl transferase
0BWT2_2717	0BWT2	2717	Terracidiphlus	GT9(109-375)	GT9	N	Glycosyl transferase
0UNS4_1758	0UNS4	1758	Terracidiphlus	GT9(91-318)	GT9	N	Glycosyl transferase
0BWT1_1953	0BWT1	1953	Terracidiphlus	GT9(92-324)	GT9	N	Glycosyl transferase
0BWT2_29	0BWT2	29	Terracidiphlus	GT9(93-317)	GT9	N	Glycosyl transferase
10BWT4_1797	10BWT4	1797	Terracidiphlus	GH51(18-507)	GH51	N	Hemicellulose
10BWT4_691	10BWT4	691	Terracidiphlus	GH51(193-705)	GH51	Y(1-28)	Hemicellulose
0BWT2_3574	0BWT2	3574	Terracidiphlus	GH51(2-339)	GH51	N	Hemicellulose
0UNS5_562	0UNS5	562	Terracidiphlus	GH51(204-652)	GH51	Y(1-22)	Hemicellulose
0UNS5_170	0UNS5	170	Terracidiphlus	GH51(206-517)	GH51	Y(1-23)	Hemicellulose
0BWT1_2128	0BWT1	2128	Terracidiphlus	GH51(208-520)	GH51	Y(1-25)	Hemicellulose
0UNS4_2943	0UNS4	2943	Terracidiphlus	GH51(212-629)	GH51	Y(1-23)	Hemicellulose
0BWT1_3046	0BWT1	3046	Terracidiphlus	GH51(262-708)	GH51	N	Hemicellulose
0BWT2_2647	0BWT2	2647	Terracidiphlus	GH51(33-551)	GH51	N	Hemicellulose
0BWT1_1084	0BWT1	1084	Terracidiphlus	GH51(36-514)	GH51	Y(1-31)	Hemicellulose
10BWT4_3543	10BWT4	3543	Terracidiphlus	GH51(37-486)	GH51	Y(1-25)	Hemicellulose
0BWT1_171	0BWT1	171	Terracidiphlus	GH51(444-877)	GH51	Y(1-65)	Hemicellulose
0UNS4_1122	0UNS4	1122	Terracidiphlus	GH53(23-340)	GH53	Y(1-23)	Hemicellulose - Arabinogalactan
0BWT1_1143	0BWT1	1143	Terracidiphlus	GH53(28-339)	GH53	Y(1-24)	Hemicellulose - Arabinogalactan
10BWT4_698	10BWT4	698	Terracidiphlus	GH43_10(107-373	GH43_10	N	hemicellulose - arabinoxylan and arabinoxylooligomers
0UNS4_2805	0UNS4	2805	Terracidiphlus	GH43_10(40-246)	GH43_10	Y(1-27)	hemicellulose - arabinoxylan and arabinoxylooligomers
0UNS4_438	0UNS4	438	Terracidiphlus	GH43_10(42-311)	GH43_10	Y(1-27)	hemicellulose - arabinoxylan and arabinoxylooligomers
0BWT2_706	0BWT2	706	Terracidiphlus	GH43_10(58-328)	GH43_10	N	hemicellulose - arabinoxylan and arabinoxylooligomers
10BWT4_143	10BWT4	143	Terracidiphlus	GH43_18(77-297)	GH43_18	Y(1-25)	hemicellulose - arabinoxylan and arabinoxylooligomers
10BWT4_2373	10BWT4	2373	Terracidiphlus	GH43_24(50-285)	GH43_24	Y(1-21)	hemicellulose - arabinoxylan and arabinoxylooligomers
0BWT2_1749	0BWT2	1749	Terracidiphlus	GH43_24(59-298)	GH43_24	Y(1-30)	hemicellulose - arabinoxylan and arabinoxylooligomers
0BWT2_1607	0BWT2	1607	Terracidiphlus	GH43_24(65-312)	GH43_24	Y(1-32)	hemicellulose - arabinoxylan and arabinoxylooligomers
0UNS4_934	0UNS4	934	Terracidiphlus	GH43_24(71-306)	GH43_24	N	hemicellulose - arabinoxylan and arabinoxylooligomers
0BWT1_2045	0BWT1	2045	Terracidiphlus	GH43_28(60-322)	GH43_28	Y(1-36)	hemicellulose - arabinoxylan and arabinoxylooligomers

0BWT2_573	0BWT2	573	Terracidiphlus	GH43_28(67-325)-	GH43_28	Y(1-34)	hemicellulose - arabinoxylan and arabinoxylooligomers
0UNS4_491	0UNS4	491	Terracidiphlus	GH43_29(35-330)	GH43_29	Y(1-23)	hemicellulose - arabinoxylan and arabinoxylooligomers
0BWT1_3045	0BWT1	3045	Terracidiphlus	GH43_3(242-564)	GH43_3	Y(1-41)	hemicellulose - arabinoxylan and arabinoxylooligomers
0UNS4_1467	0UNS4	1467	Terracidiphlus	GH43_5(34-335)	GH43_5	Y(1-22)	hemicellulose - arabinoxylan and arabinoxylooligomers
0BWT1_3008	0BWT1	3008	Terracidiphlus	GH43_5(36-337)	GH43_5	Y(1-27)	hemicellulose - arabinoxylan and arabinoxylooligomers
10BWT4_1491	10BWT4	1491	Terracidiphlus	GH43_5(39-341)	GH43_5	Y(1-24)	hemicellulose - arabinoxylan and arabinoxylooligomers
0UNS4_223	0UNS4	223	Terracidiphlus	GH43_9(32-296)	GH43_9	Y(1-28)	hemicellulose - arabinoxylan and arabinoxylooligomers
0UNS4_2804	0UNS4	2804	Terracidiphlus	GH43_9(43-304)	GH43_19+GH4	N	hemicellulose - arabinoxylan and arabinoxylooligomers
0BWT1_3507	0BWT1	3507	Terracidiphlus	GH54(41-368)	GH54	Y(1-25)	Hemicellulose - Arabinoxylans and arabinogalactans
0BWT1_2710	0BWT1	2710	Terracidiphlus	GH54(79-388)+CBI	CBM42+GH54	N	Hemicellulose - Arabinoxylans and arabinogalactans
0UNS4_1196	0UNS4	1196	Terracidiphlus	PL8_3(350-591)	PL8	N	hyaluronan
0BWT2_4313	0BWT2	4313	Terracidiphlus	GH136(1-317)	GH136	N	lacto-N-tetraose (cleaves lacto-N-biose)
0BWT1_1968	0BWT1	1968	Terracidiphlus	GH136(23-405)	GH136	Y(1-23)	lacto-N-tetraose (cleaves lacto-N-biose)
0BWT1_1459	0BWT1	1459	Terracidiphlus	GH136(23-448)	GH136	Y(1-23)	lacto-N-tetraose (cleaves lacto-N-biose)
10BWT4_2732	10BWT4	2732	Terracidiphlus	CE15(109-368)	CE15	N	Lignocellulose
0BWT2_711	0BWT2	711	Terracidiphlus	CE15(36-430)	CE15	Y(1-26)	Lignocellulose
0UNS4_1197	0UNS4	1197	Terracidiphlus	CE15(60-438)	CE15	Y(1-24)	Lignocellulose
10BWT4_2240	10BWT4	2240	Terracidiphlus	CE15(62-445)	CE15	N	Lignocellulose
10BWT4_3536	10BWT4	3536	Terracidiphlus	CE15(88-471)	CE15	Y(1-40)	Lignocellulose
0UNS5_2402	0UNS5	2402	Terracidiphlus	CE15(90-468)	CE15	Y(1-35)	Lignocellulose
0BWT2_483	0BWT2	483	Terracidiphlus	CE15(95-472)	CE15	Y(1-32)	Lignocellulose
0UNS5_1188	0UNS5	1188	Terracidiphlus	GH125(68-441)	GH125	Y(1-29)	mannose cleavage
0BWT1_3405	0BWT1	3405	Terracidiphlus	GH125(68-470)	GH125	Y(1-28)	mannose cleavage
0UNS4_45	0UNS4	45	Terracidiphlus	GH125(73-472)	GH125	Y(1-29)	mannose cleavage
10BWT4_3132	10BWT4	3132	Terracidiphlus	GH125(84-347)	GH125	N	mannose cleavage
0BWT2_4208	0BWT2	4208	Terracidiphlus	GH38(2-194)	GH38	N	mannose cleavage
10BWT4_481	10BWT4	481	Terracidiphlus	GH38(262-518)	GH38	Y(1-22)	mannose cleavage
0BWT1_2747	0BWT1	2747	Terracidiphlus	GH38(264-518)	GH38	N	mannose cleavage
0BWT2_1393	0BWT2	1393	Terracidiphlus	GH38(267-441)	GH38	Y(1-20)	mannose cleavage
0BWT2_717	0BWT2	717	Terracidiphlus	GH38(270-522)	GH38	Y(1-42)	mannose cleavage
10BWT4_406	10BWT4	406	Terracidiphlus	GH38(273-535)	GH38	Y(1-29)	mannose cleavage
0BWT1_2730	0BWT1	2730	Terracidiphlus	GH38(70-300)	GH38	N	mannose cleavage
10BWT4_3331	10BWT4	3331	Terracidiphlus	GH92(2-235)	GH92	N	mannose cleavage
10BWT4_2515	10BWT4	2515	Terracidiphlus	GH92(2-442)	GH92	N	mannose cleavage
0BWT2_948	0BWT2	948	Terracidiphlus	GH92(222-742)	GH92	Y(1-20)	mannose cleavage
10BWT4_1368	10BWT4	1368	Terracidiphlus	GH92(239-733)	GH92	Y(1-26)	mannose cleavage
0UNS4_2642	0UNS4	2642	Terracidiphlus	GH92(241-721)	GH92	Y(1-26)	mannose cleavage
0BWT2_1862	0BWT2	1862	Terracidiphlus	GH92(241-734)	GH92	Y(1-22)	mannose cleavage
0BWT1_636	0BWT1	636	Terracidiphlus	GH92(245-737)	GH92	Y(1-25)	mannose cleavage
0UNS5_88	0UNS5	88	Terracidiphlus	GH92(252-732)	GH92	N	mannose cleavage
0BWT1_2727	0BWT1	2727	Terracidiphlus	GH92(253-742)	GH92	Y(1-34)	mannose cleavage
10BWT4_1248	10BWT4	1248	Terracidiphlus	GH92(254-626)	GH92	Y(1-19)	mannose cleavage
0BWT2_3961	0BWT2	3961	Terracidiphlus	GH92(26-524)	GH92	N	mannose cleavage
0UNS4_2940	0UNS4	2940	Terracidiphlus	GH92(261-751)	GH92	Y(1-19)	mannose cleavage
0UNS5_167	0UNS5	167	Terracidiphlus	GH92(261-753)	GH92	Y(1-22)	mannose cleavage
0BWT2_944	0BWT2	944	Terracidiphlus	GH92(262-759)	GH92	Y(1-27)	mannose cleavage
0UNS4_1243	0UNS4	1243	Terracidiphlus	GH92(263-760)	GH92	Y(1-33)	mannose cleavage
0UNS5_815	0UNS5	815	Terracidiphlus	GH92(263-760)	GH92	Y(1-43)	mannose cleavage
0BWT1_3120	0BWT1	3120	Terracidiphlus	GH92(268-765)	GH92	Y(1-40)	mannose cleavage
0BWT1_2731	0BWT1	2731	Terracidiphlus	GH92(291-777)	GH92	Y(1-27)	mannose cleavage
0BWT2_2151	0BWT2	2151	Terracidiphlus	GH92(305-792)	GH92	N	mannose cleavage
0BWT2_3644	0BWT2	3644	Terracidiphlus	GH92(76-584)	GH92	N	mannose cleavage
10BWT4_185	10BWT4	185	Terracidiphlus	GH27(118-334)	GH27	N	Oligosaccharides (melibiose, raffinose, and stachyose)
0BWT2_2144	0BWT2	2144	Terracidiphlus	GH27(175-359)	GH27	Y(1-17)	Oligosaccharides (melibiose, raffinose, and stachyose)
0BWT2_1156	0BWT2	1156	Terracidiphlus	GH27(186-373)	GH27	Y(1-24)	Oligosaccharides (melibiose, raffinose, and stachyose)
0UNS5_137	0UNS5	137	Terracidiphlus	GH27(20-199)+GH	GH27	Y(1-22)	Oligosaccharides (melibiose, raffinose, and stachyose)
0BWT1_1377	0BWT1	1377	Terracidiphlus	GH27(26-200)+GH	GH27	Y(1-23)	Oligosaccharides (melibiose, raffinose, and stachyose)
10BWT4_3287	10BWT4	3287	Terracidiphlus	GH27(26-210)+GH	GH27	Y(1-31)	Oligosaccharides (melibiose, raffinose, and stachyose)
0BWT1_2079	0BWT1	2079	Terracidiphlus	GH27(43-227)	GH27	N	Oligosaccharides (melibiose, raffinose, and stachyose)
0BWT2_473	0BWT2	473	Terracidiphlus	CE12(172-374)	CE12	Y(1-19)	Pectin
0UNS4_1686	0UNS4	1686	Terracidiphlus	CE12(174-376)	CE12	Y(1-19)	Pectin
0UNS5_1197	0UNS5	1197	Terracidiphlus	CE12(174-377)	CE12	Y(1-29)	Pectin
0UNS4_1596	0UNS4	1596	Terracidiphlus	CE12(31-227)+CE1	CE12	Y(1-21)	Pectin
0UNS5_631	0UNS5	631	Terracidiphlus	CE12(31-227)+CE1	CE12	Y(1-21)	Pectin
0BWT2_490	0BWT2	490	Terracidiphlus	CE12(37-232)+CE1	CE12	Y(1-22)	Pectin
0BWT1_1451	0BWT1	1451	Terracidiphlus	CE12(529-726)	CBM35	Y(1-33)	Pectin
0BWT2_470	0BWT2	470	Terracidiphlus	CE12(68-286)	CE12	Y(1-23)	Pectin
0UNS4_1656	0UNS4	1656	Terracidiphlus	CE8(1267-1618)+C	GH28	Y(1-26)	Pectin

0BWT2_254	0BWT2	254	Terracidiphilus	CE8(34-327)	CE8	Y(1-19)	Pectin
10BWT4_2718	10BWT4	2718	Terracidiphilus	CE8(42-328)	CE8	Y(1-27)	Pectin
10BWT4_2335	10BWT4	2335	Terracidiphilus	CE8(65-248)	CBM50	Y(1-28)	Pectin
0UNS5_1875	0UNS5	1875	Terracidiphilus	CE8(706-1057)+GH	GH28	N	Pectin
10BWT4_2976	10BWT4	2976	Terracidiphilus	GH106(1-440)	GH106	N	Pectin
0BWT2_3539	0BWT2	3539	Terracidiphilus	GH106(126-854)	GH106	N	Pectin
0UNS5_104	0UNS5	104	Terracidiphilus	GH106(198-996)	GH106	N	Pectin
0UNS4_1972	0UNS4	1972	Terracidiphilus	GH106(213-1016)	GH106	Y(1-25)	Pectin
0BWT2_1186	0BWT2	1186	Terracidiphilus	GH106(241-961)	GH106	N	Pectin
10BWT4_3039	10BWT4	3039	Terracidiphilus	GH106(315-923)	GH106	N	Pectin
10BWT4_774	10BWT4	774	Terracidiphilus	GH106(34-907)	GH106	Y(1-19)	Pectin
0BWT2_1748	0BWT2	1748	Terracidiphilus	GH106(370-926)	GH106	N	Pectin
0UNS5_184	0UNS5	184	Terracidiphilus	GH106(379-1111)	GH106	Y(1-23)	Pectin
0UNS4_1566	0UNS4	1566	Terracidiphilus	GH106(38-735)	GH106	N	Pectin
0BWT2_703	0BWT2	703	Terracidiphilus	GH106(38-742)	GH106	Y(1-24)	Pectin
0BWT1_3525	0BWT1	3525	Terracidiphilus	GH106(389-1115)	GH106	N	Pectin
0UNS4_2944	0UNS4	2944	Terracidiphilus	GH106(40-795)	GH106	Y(1-30)	Pectin
0UNS5_171	0UNS5	171	Terracidiphilus	GH106(40-795)	GH106	Y(1-30)	Pectin
0BWT2_472	0BWT2	472	Terracidiphilus	GH106(42-804)	GH106	Y(1-36)	Pectin
0BWT1_900	0BWT1	900	Terracidiphilus	GH106(42-916)	GH106	N	Pectin
10BWT4_1845	10BWT4	1845	Terracidiphilus	GH106(57-976)	GH106	Y(1-33)	Pectin
0BWT2_2734	0BWT2	2734	Terracidiphilus	GH106(58-734)	GH106	N	Pectin
10BWT4_2961	10BWT4	2961	Terracidiphilus	GH137(43-362)	GH137	Y(1-28)	Pectin
10BWT4_142	10BWT4	142	Terracidiphilus	GH138(11-885)	GH138	Y(1-28)	Pectin
0BWT2_2150	0BWT2	2150	Terracidiphilus	GH139(25-709)	GH139	Y(1-38)	Pectin
10BWT4_145	10BWT4	145	Terracidiphilus	GH142(54-527)	GH142	Y(1-27)	Pectin
0BWT1_455	0BWT1	455	Terracidiphilus	GH142(59-543)	GH142	Y(1-39)	Pectin
10BWT4_1920	10BWT4	1920	Terracidiphilus	GH146(43-537)	GH146	N	Pectin
0BWT2_3732	0BWT2	3732	Terracidiphilus	GH146(48-554)	GH146	Y(1-29)	Pectin
0BWT1_3030	0BWT1	3030	Terracidiphilus	GH146(54-562)	GH146	Y(1-31)	Pectin
0UNS5_2760	0UNS5	2760	Terracidiphilus	GH146(60-356)	GH146	Y(1-31)	Pectin
0UNS4_490	0UNS4	490	Terracidiphilus	GH146(60-556)	GH146	Y(1-31)	Pectin
10BWT4_2685	10BWT4	2685	Terracidiphilus	GH146(63-554)	GH146	Y(1-33)	Pectin
0BWT1_1085	0BWT1	1085	Terracidiphilus	GH146(63-573)	GH146	Y(1-20)	Pectin
0UNS4_1242	0UNS4	1242	Terracidiphilus	GH146(79-581)	GH146	Y(1-36)	Pectin
10BWT4_1527	10BWT4	1527	Terracidiphilus	GH146(88-591)	GH146	Y(1-35)	Pectin
0BWT2_720	0BWT2	720	Terracidiphilus	GH146(95-500)	GH146	N	Pectin
10BWT4_891	10BWT4	891	Terracidiphilus	GH28(1-192)	GH28	N	Pectin
0UNS5_1574	0UNS5	1574	Terracidiphilus	GH28(1-252)	GH28	N	Pectin
0UNS4_1595	0UNS4	1595	Terracidiphilus	GH28(109-405)+CE	CE8+GH28	Y(1-26)	Pectin
0UNS5_632	0UNS5	632	Terracidiphilus	GH28(109-412)+CE	CE8+GH28	Y(1-63)	Pectin
10BWT4_3168	10BWT4	3168	Terracidiphilus	GH28(11-290)	GH28	N	Pectin
0BWT1_2953	0BWT1	2953	Terracidiphilus	GH28(122-440)	GH28	Y(1-31)	Pectin
0BWT2_742	0BWT2	742	Terracidiphilus	GH28(1316-1646)	GH28	Y(1-26)	Pectin
0BWT2_704	0BWT2	704	Terracidiphilus	GH28(136-439)	GH28	Y(1-31)	Pectin
0BWT1_3078	0BWT1	3078	Terracidiphilus	GH28(15-311)	GH28	N	Pectin
0UNS5_40	0UNS5	40	Terracidiphilus	GH28(176-438)	GH28	Y(1-23)	Pectin
0BWT2_149	0BWT2	149	Terracidiphilus	GH28(177-443)	GH28	Y(1-21)	Pectin
0UNS5_2695	0UNS5	2695	Terracidiphilus	GH28(2-286)	GH28	N	Pectin
0BWT2_642	0BWT2	642	Terracidiphilus	GH28(52-387)	GH28	Y(1-22)	Pectin
10BWT4_2184	10BWT4	2184	Terracidiphilus	GH28(53-424)	GH28	Y(1-22)	Pectin
0UNS4_1959	0UNS4	1959	Terracidiphilus	GH28(54-395)	GH28	Y(1-27)	Pectin
0UNS5_2735	0UNS5	2735	Terracidiphilus	GH28(54-423)	GH28	Y(1-24)	Pectin
0UNS4_2029	0UNS4	2029	Terracidiphilus	GH28(55-376)	GH28	N	Pectin
0UNS5_87	0UNS5	87	Terracidiphilus	GH28(55-376)	GH28	N	Pectin
0UNS5_662	0UNS5	662	Terracidiphilus	GH28(55-390)	GH28	Y(1-27)	Pectin
10BWT4_3162	10BWT4	3162	Terracidiphilus	GH28(55-402)	GH28	Y(1-28)	Pectin
0UNS4_1947	0UNS4	1947	Terracidiphilus	GH28(55-423)	GH28	Y(1-24)	Pectin
0UNS4_2068	0UNS4	2068	Terracidiphilus	GH28(56-395)	GH28	Y(1-29)	Pectin
0UNS5_93	0UNS5	93	Terracidiphilus	GH28(56-398)	GH28	Y(1-29)	Pectin
0BWT2_471	0BWT2	471	Terracidiphilus	GH28(56-429)	GH28	Y(1-25)	Pectin
0BWT1_714	0BWT1	714	Terracidiphilus	GH28(57-396)	GH28	Y(1-23)	Pectin
0BWT2_266	0BWT2	266	Terracidiphilus	GH28(59-397)	GH28	Y(1-29)	Pectin
0BWT2_1864	0BWT2	1864	Terracidiphilus	GH28(61-416)	GH28	Y(1-31)	Pectin
0UNS5_2602	0UNS5	2602	Terracidiphilus	GH28(69-391)	GH28	Y(1-39)	Pectin
0UNS4_2058	0UNS4	2058	Terracidiphilus	GH28(71-396)	GH28	Y(1-29)	Pectin
0UNS4_194	0UNS4	194	Terracidiphilus	GH28(71-432)	GH28	Y(1-31)	Pectin
0UNS4_1464	0UNS4	1464	Terracidiphilus	GH28(72-392)	GH28	Y(1-23)	Pectin

0UNS4_2839	0UNS4	2839	Terracidiphlus	GH28(72-437)	GH28	Y(1-31)	Pectin
0UNS5_2098	0UNS5	2098	Terracidiphlus	GH28(73-394)	GH28	Y(1-25)	Pectin
0BWT2_3050	0BWT2	3050	Terracidiphlus	GH28(73-433)	GH28	Y(1-34)	Pectin
0BWT2_1645	0BWT2	1645	Terracidiphlus	GH28(79-405)	GH28	Y(1-22)	Pectin
0BWT2_1959	0BWT2	1959	Terracidiphlus	GH28(83-398)	GH28	N	Pectin
10BWT4_184	10BWT4	184	Terracidiphilus	GH28(83-434)	GH28	N	Pectin
0BWT2_491	0BWT2	491	Terracidiphlus	GH28(85-401)+CE8	CE8+GH28	Y(1-24)	Pectin
10BWT4_2720	10BWT4	2720	Terracidiphilus	GH28(860-1177)	GH28	N	Pectin
0BWT2_690	0BWT2	690	Terracidiphlus	GH28(87-402)	GH28	Y(1-22)	Pectin
0BWT2_489	0BWT2	489	Terracidiphlus	GH28(88-397)+CE8	CE8+GH28	Y(1-23)	Pectin
0BWT1_704	0BWT1	704	Terracidiphlus	GH28(89-396)+CE8	CE8+GH28	Y(1-24)	Pectin
0BWT2_2612	0BWT2	2612	Terracidiphlus	GH28(90-435)	GH28	Y(1-35)	Pectin
0UNS4_179	0UNS4	179	Terracidiphlus	GH28(96-416)	GH28	Y(1-32)	Pectin
10BWT4_2241	10BWT4	2241	Terracidiphilus	GH28(97-423)	GH28	Y(1-49)	Pectin
0UNS4_1968	0UNS4	1968	Terracidiphlus	PL10_1(119-422)	PL10_1	Y(1-20)	Pectin
0BWT2_487	0BWT2	487	Terracidiphlus	PL10_1(122-423)	PL10_1	Y(1-28)	Pectin
10BWT4_2032	10BWT4	2032	Terracidiphilus	PL10_1(122-433)	PL10_1	Y(1-28)	Pectin
0UNS5_364	0UNS5	364	Terracidiphlus	PL22_2(216-440)	PL22_2	Y(1-20)	Pectin
0BWT2_474	0BWT2	474	Terracidiphlus	PL22_2(219-439)	PL22_2	Y(1-22)	Pectin
0UNS4_920	0UNS4	920	Terracidiphlus	PL22_2(223-447)	PL22_2	Y(1-21)	Pectin
0UNS4_1366	0UNS4	1366	Terracidiphlus	PL4_1(28-560)	PL4	Y(1-30)	Pectin
0UNS5_2694	0UNS5	2694	Terracidiphlus	PL4(2-365)	PL4	N	Pectin
0BWT2_1956	0BWT2	1956	Terracidiphlus	GH141(24-568)	GH141	Y(1-34)	Pectin and xylan
10BWT4_1487	10BWT4	1487	Terracidiphilus	GH23(215-350)	CBM50+GH23	Y(1-22)	peptidoglycan, chitin and chitooligosaccharides
0UNS4_1021	0UNS4	1021	Terracidiphlus	GH23(222-353)	CBM50+GH23	Y(1-40)	peptidoglycan, chitin and chitooligosaccharides
0BWT1_2113	0BWT1	2113	Terracidiphlus	GH23(223-349)	CBM50+GH23	Y(1-34)	peptidoglycan, chitin and chitooligosaccharides
0BWT2_47	0BWT2	47	Terracidiphlus	GH23(225-351)	CBM50+GH23	Y(1-32)	peptidoglycan, chitin and chitooligosaccharides
0UNS5_49	0UNS5	49	Terracidiphlus	GH23(228-355)	CBM50+GH23	Y(1-41)	peptidoglycan, chitin and chitooligosaccharides
0BWT2_2788	0BWT2	2788	Terracidiphlus	GH23(695-834)	GH23	Y(1-32)	peptidoglycan, chitin and chitooligosaccharides
0UNS4_1688	0UNS4	1688	Terracidiphlus	GH23(719-856)	GH23	Y(1-36)	peptidoglycan, chitin and chitooligosaccharides
0BWT1_1773	0BWT1	1773	Terracidiphlus	GH23(769-907)	GH23	Y(1-41)	peptidoglycan, chitin and chitooligosaccharides
0UNS5_1195	0UNS5	1195	Terracidiphlus	GH23(788-924)	GH23	Y(1-34)	peptidoglycan, chitin and chitooligosaccharides
0BWT1_2495	0BWT1	2495	Terracidiphlus	AA12(102-468)	N	Y(1-31)	PQQ-dependent oxidoreductase
10AWT2_2071	10AWT2	2071	Holophaga	AA6(4-183)	N	N	quinone reduction
10BWT3_1331	10BWT3	1331	Holophaga	AA6(4-183)	N	N	quinone reduction
10UNS1_121	10UNS1	121	Holophaga	AA6(4-183)	N	N	quinone reduction
5AWT2_3115	5AWT2	3115	Holophaga	AA6(4-183)	N	N	quinone reduction
5BWT7_2569	5BWT7	2569	Holophaga	AA6(4-183)	N	N	quinone reduction
5UNS4_3266	5UNS4	3266	Holophaga	AA6(4-183)	N	N	quinone reduction
0BWT2_2357	0BWT2	2357	Terracidiphlus	CBM67(14-107)+CI	CBM67+GH78	N	Rhamnose cleavage
0BWT2_2440	0BWT2	2440	Terracidiphlus	CBM67(143-311)+	GH78	N	Rhamnose cleavage
0BWT1_2072	0BWT1	2072	Terracidiphlus	CBM67(175-346)+	GH78	Y(1-25)	Rhamnose cleavage
10BWT4_3221	10BWT4	3221	Terracidiphilus	CBM67(2-160)+CBI	CBM67+GH78	N	Rhamnose cleavage
0BWT1_2723	0BWT1	2723	Terracidiphlus	CBM67(42-198)+G	CBM67+GH78	Y(1-36)	Rhamnose cleavage
0UNS5_565	0UNS5	565	Terracidiphlus	GH78(107-559)	GH78	Y(1-30)	rhamnoside cleavage
0BWT1_950	0BWT1	950	Terracidiphlus	GH78(347-686)	GH0	N	rhamnoside cleavage
10BWT4_505	10BWT4	505	Terracidiphilus	GH78(46-555)	GH78	N	rhamnoside cleavage
10AWT2_1554	10AWT2	1554	Holophaga	CBM48(10-103)+G	GH13_10+GH1	N	Starch
5AWT2_3796	5AWT2	3796	Holophaga	CBM48(10-103)+G	GH13_10+GH1	N	Starch
5UNS4_1484	5UNS4	1484	Holophaga	CBM48(10-103)+G	GH13_10+GH1	N	Starch
10BWT3_4460	10BWT3	4460	Holophaga	CBM48(122-205)+	CBM48+GH13_	N	Starch
10AWT2_2649	10AWT2	2649	Holophaga	CBM48(122-205)+	CBM48+GH13_	N	Starch
10UNS1_3144	10UNS1	3144	Holophaga	CBM48(122-205)+	CBM48+GH13_	N	Starch
5AWT2_2774	5AWT2	2774	Holophaga	CBM48(122-205)+	CBM48+GH13_	N	Starch
5BWT7_1938	5BWT7	1938	Holophaga	CBM48(122-205)+	CBM48+GH13_	N	Starch
5UNS4_2188	5UNS4	2188	Holophaga	CBM48(122-205)+	CBM48+GH13_	N	Starch
5AWT5_2295	5AWT5	2295	Holophaga	CBM48(125-211)	CBM48+GH13_	N	Starch
10AWT2_1907	10AWT2	1907	Holophaga	CBM48(130-216)+	CBM48+GH13_	N	Starch
10BWT3_2701	10BWT3	2701	Holophaga	CBM48(130-216)+	CBM48+GH13_	N	Starch
5AWT2_300	5AWT2	300	Holophaga	CBM48(130-216)+	CBM48+GH13_	N	Starch
5BWT7_1469	5BWT7	1469	Holophaga	CBM48(130-216)+	CBM48+GH13_	N	Starch
5UNS4_2640	5UNS4	2640	Holophaga	CBM48(130-216)+	CBM48+GH13_	N	Starch
10UNS1_3537	10UNS1	3537	Holophaga	GH13_11(1-303)	GH13_10+GH1	N	Starch
5BWT7_4100	5BWT7	4100	Holophaga	GH13_11(1-332)	GH13_10+GH1	N	Starch
5AWT5_1914	5AWT5	1914	Holophaga	GH13_11(177-530	CBM48+GH13_	N	Starch
0UNS4_1605	0UNS4	1605	Terracidiphlus	GH13(154-471)	GH13	Y(1-21)	Starch
0BWT2_1243	0BWT2	1243	Terracidiphlus	GH13(162-479)	GH13	Y(1-22)	Starch
0BWT2_3901	0BWT2	3901	Terracidiphlus	GH13(162-479)	GH13	Y(1-23)	Starch

0BWT1_2262	0BWT1	2262	Terracidiphlus	GH13(163-480)	GH13	Y(1-25)	Starch
0UNS5_2348	0UNS5	2348	Terracidiphlus	GH13(174-451)	GH13	Y(1-21)	Starch
10BWT4_846	10BWT4	846	Terracidiphilus	GH13(336-707)	GH13	N	Starch
0UNS5_159	0UNS5	159	Terracidiphlus	GH13(337-708)	GH13	N	Starch
0BWT1_3147	0BWT1	3147	Terracidiphlus	GH13(343-704)	GH13	N	Starch
0UNS4_2070	0UNS4	2070	Terracidiphlus	GH13(345-705)	GH13	N	Starch
0BWT2_2215	0BWT2	2215	Terracidiphlus	GH13(348-708)	GH13	N	Starch
0BWT1_2263	0BWT1	2263	Terracidiphlus	GH13_2(261-591)	CBM20+GH13_	Y(1-39)	Starch, glycogen
10BWT4_1282	10BWT4	1282	Terracidiphilus	GH13_2(66-380)+(	CBM20+GH13_	Y(1-22)	Starch, glycogen
10BWT4_3377	10BWT4	3377	Terracidiphilus	GH31(2-390)	GH31	N	Starch, glycoprotein
0UNS4_2546	0UNS4	2546	Terracidiphlus	GH31(207-641)	GH31	Y(1-20)	Starch, glycoprotein
0BWT2_2438	0BWT2	2438	Terracidiphlus	GH31(215-643)	GH31	Y(1-30)	Starch, glycoprotein
10BWT4_2695	10BWT4	2695	Terracidiphilus	GH31(216-644)	GH31	Y(1-33)	Starch, glycoprotein
0BWT1_2735	0BWT1	2735	Terracidiphlus	GH31(221-666)	CBM35+GH31	Y(1-24)	Starch, glycoprotein
0UNS5_1974	0UNS5	1974	Terracidiphlus	GH31(222-656)	GH31	Y(1-38)	Starch, glycoprotein
0BWT1_643	0BWT1	643	Terracidiphlus	GH31(225-661)	GH31	Y(1-24)	Starch, glycoprotein
0BWT2_3541	0BWT2	3541	Terracidiphlus	GH31(226-677)	GH31	Y(1-19)	Starch, glycoprotein
0BWT1_1666	0BWT1	1666	Terracidiphlus	GH31(237-716)	GH31	Y(1-29)	Starch, glycoprotein
10BWT4_2383	10BWT4	2383	Terracidiphilus	GH31(239-659)	GH31	Y(1-27)	Starch, glycoprotein
10BWT4_715	10BWT4	715	Terracidiphilus	GH31(244-720)	GH31	Y(1-29)	Starch, glycoprotein
0BWT2_1281	0BWT2	1281	Terracidiphlus	GH31(245-722)	GH31	Y(1-43)	Starch, glycoprotein
0BWT1_1721	0BWT1	1721	Terracidiphlus	GH31(247-680)	GH31	Y(1-47)	Starch, glycoprotein
0BWT2_2302	0BWT2	2302	Terracidiphlus	GH31(247-717)	GH31	Y(1-24)	Starch, glycoprotein
0UNS4_1920	0UNS4	1920	Terracidiphlus	GH31(257-713)	GH31	N	Starch, glycoprotein
0UNS4_1391	0UNS4	1391	Terracidiphlus	GH31(258-691)	GH31	N	Starch, glycoprotein
0UNS5_335	0UNS5	335	Terracidiphlus	GH31(260-716)	GH31	Y(1-24)	Starch, glycoprotein
0BWT1_88	0BWT1	88	Terracidiphlus	GH31(284-735)	GH31	N	Starch, glycoprotein
10AWT2_3241	10AWT2	3241	Holophaga	GH31(299-523)	GH31	N	Starch, glycoprotein
10BWT3_216	10BWT3	216	Holophaga	GH31(299-523)	GH31	N	Starch, glycoprotein
10UNS1_3125	10UNS1	3125	Holophaga	GH31(299-523)	GH31	N	Starch, glycoprotein
5AWT2_2985	5AWT2	2985	Holophaga	GH31(299-523)	GH31	N	Starch, glycoprotein
5BWT7_4125	5BWT7	4125	Holophaga	GH31(299-523)	GH31	N	Starch, glycoprotein
5UNS4_741	5UNS4	741	Holophaga	GH31(299-523)	GH31	N	Starch, glycoprotein
0BWT1_759	0BWT1	759	Terracidiphlus	GH31(365-822)	GH31	Y(1-20)	Starch, glycoprotein
10BWT4_108	10BWT4	108	Terracidiphilus	GH31(369-826)	GH31	Y(1-23)	Starch, glycoprotein
0UNS4_2249	0UNS4	2249	Terracidiphlus	GH57(45-333)	GH57	N	starch, pullalan
0UNS5_214	0UNS5	214	Terracidiphlus	GH57(46-330)	GH57	N	starch, pullalan
10BWT4_2023	10BWT4	2023	Terracidiphilus	GH57(55-337)	GH57	N	starch, pullalan
0BWT2_2083	0BWT2	2083	Terracidiphlus	GH57(56-332)	GH57	N	starch, pullalan
0BWT1_104	0BWT1	104	Terracidiphlus	GH57(57-331)	GH57	N	starch, pullalan
0BWT1_3062	0BWT1	3062	Terracidiphlus	GH13_18(53-396)	GH13_18	N	sucrose conversions not hydrolysis
10BWT4_1823	10BWT4	1823	Terracidiphilus	GH37(77-549)	GH37	Y(1-24)	trehalose
0BWT2_2340	0BWT2	2340	Terracidiphlus	GH37(79-543)	GH37	Y(1-22)	trehalose
5AWT5_873	5AWT5	873	Holophaga	GH13_10(124-436	CBM48+GH13_	N	Trehalose biosynthesis
10AWT2_1555	10AWT2	1555	Holophaga	GH13_10(125-439	CBM48+GH13_	N	Trehalose biosynthesis
10BWT3_2806	10BWT3	2806	Holophaga	GH13_10(125-439	CBM48+GH13_	N	Trehalose biosynthesis
5AWT2_3795	5AWT2	3795	Holophaga	GH13_10(125-439	CBM48+GH13_	N	Trehalose biosynthesis
5UNS4_1485	5UNS4	1485	Holophaga	GH13_10(125-439	CBM48+GH13_	N	Trehalose biosynthesis
10UNS1_3536	10UNS1	3536	Holophaga	GH13_10(125-439	GH13_10	N	Trehalose biosynthesis
5BWT7_4101	5BWT7	4101	Holophaga	GH13_10(125-439	GH13_10	N	Trehalose biosynthesis
5AWT5_872	5AWT5	872	Holophaga	GH13_16(2-198)	GH13_16	N	Trehalose biosynthesis
10BWT4_77	10BWT4	77	Terracidiphilus	GH13_16(35-388)	GH13_16	N	Trehalose biosynthesis
5AWT5_882	5AWT5	882	Holophaga	GH15(168-529)	GH15	N	trehalose, starch, dextran
10AWT2_4017	10AWT2	4017	Holophaga	GH15(19-262)	GH15	N	trehalose, starch, dextran
0BWT1_825	0BWT1	825	Terracidiphlus	GH15(216-577)	GH15	N	trehalose, starch, dextran
10UNS1_1628	10UNS1	1628	Holophaga	GH15(225-585)	GH15	N	trehalose, starch, dextran
5AWT2_2503	5AWT2	2503	Holophaga	GH15(225-585)	GH15	N	trehalose, starch, dextran
5BWT7_853	5BWT7	853	Holophaga	GH15(225-585)	GH15	N	trehalose, starch, dextran
5UNS4_3100	5UNS4	3100	Holophaga	GH15(225-585)	GH15	N	trehalose, starch, dextran
5AWT5_3055	5AWT5	3055	Holophaga	GH15(296-683)	GH15	N	trehalose, starch, dextran
0BWT2_2254	0BWT2	2254	Terracidiphlus	GH15(303-691)	GH15	N	trehalose, starch, dextran
10BWT4_2506	10BWT4	2506	Terracidiphilus	GH15(323-711)	GH15	N	trehalose, starch, dextran
10UNS1_4437	10UNS1	4437	Holophaga	GH13_23(1-182)	GH13_23	N	Unknown
10UNS1_4541	10UNS1	4541	Holophaga	GH13_23(39-329)	GH13_23	N	Unknown
10AWT2_1062	10AWT2	1062	Holophaga	GH13_23(39-389)	GH13_23	N	Unknown
10BWT3_1161	10BWT3	1161	Holophaga	GH13_23(39-389)	GH13_23	N	Unknown
5AWT2_1486	5AWT2	1486	Holophaga	GH13_23(39-389)	GH13_23	N	Unknown
5BWT7_336	5BWT7	336	Holophaga	GH13_23(39-389)	GH13_23	N	Unknown

5UNS4_2206	5UNS4	2206	Holophaga	GH13_23(39-389)	GH13_23	N	Unknown
0BWT1_951	0BWT1	951	Terracidiphlus	GH13_23(68-404)	GH13_23	Y(1-36)	Unknown
0BWT2_2362	0BWT2	2362	Terracidiphlus	GH13_23(69-427)	GH13_23	Y(1-25)	Unknown
0UNS5_396	0UNS5	396	Terracidiphlus	GH13_23(82-440)	GH13_23	Y(1-29)	Unknown
0UNS4_886	0UNS4	886	Terracidiphlus	GH13_23(84-442)	GH13_23	Y(1-30)	Unknown
5AWT5_2296	5AWT5	2296	Holophaga	GH13_3(185-381)-	GH133+GH13	N	Unknown
10BWT3_2901	10BWT3	2901	Holophaga	GH13_3(190-384)	GH133+GH13	N	Unknown
10AWT2_1906	10AWT2	1906	Holophaga	GH13_3(190-384)-	GH133+GH13	N	Unknown
10UNS1_1280	10UNS1	1280	Holophaga	GH13_3(190-384)-	GH133+GH13	N	Unknown
5AWT2_301	5AWT2	301	Holophaga	GH13_3(190-384)-	GH133+GH13	N	Unknown
5BWT7_1468	5BWT7	1468	Holophaga	GH13_3(190-384)-	GH133+GH13	N	Unknown
5UNS4_2641	5UNS4	2641	Holophaga	GH13_3(190-384)-	GH133+GH13	N	Unknown
5AWT5_1758	5AWT5	1758	Holophaga	AA4(37-233)	N	N	Vanillin alcohol to vanillin; phenolic compounds
0BWT2_1822	0BWT2	1822	Terracidiphlus	GH10(27-366)	GH10	Y(1-21)	Xylan
10BWT4_23	10BWT4	23	Terracidiphilus	GH10(36-377)	GH10	Y(1-31)	Xylan
0UNS4_475	0UNS4	475	Terracidiphlus	GH115(49-868)	GH115	N	Xylan
0BWT2_709	0BWT2	709	Terracidiphlus	GH115(5-324)	GH115	N	Xylan
0UNS5_1120	0UNS5	1120	Terracidiphlus	GH115(50-884)	GH115	Y(1-35)	Xylan
0BWT2_1246	0BWT2	1246	Terracidiphlus	GH115(57-329)	GH115	Y(1-26)	Xylan
0BWT1_1453	0BWT1	1453	Terracidiphlus	GH115(61-892)	GH115	N	Xylan
10BWT3_4114	10BWT3	4114	Holophaga	GH30_2(41-403)	GH30_2	N	Xylan
10AWT2_184	10AWT2	184	Holophaga	GH30_2(41-446)	GH30_2	N	Xylan
10UNS1_776	10UNS1	776	Holophaga	GH30_2(41-446)	GH30_2	N	Xylan
5AWT2_778	5AWT2	778	Holophaga	GH30_2(41-446)	GH30_2	N	Xylan
5UNS4_490	5UNS4	490	Holophaga	GH30_2(41-446)	GH30_2	N	Xylan
0BWT1_1119	0BWT1	1119	Terracidiphlus	GH30_2(82-494)	GH30_2	Y(1-35)	Xylan
0UNS5_564	0UNS5	564	Terracidiphlus	GH30_2(89-499)	GH30_2	N	Xylan
0BWT1_1976	0BWT1	1976	Terracidiphlus	GH30_3(317-735)-	CBM61+CBM6	Y(1-28)	Xylan
0BWT2_1354	0BWT2	1354	Terracidiphlus	GH30_3(478-896)-	CBM61+CBM6	Y(1-34)	Xylan
0UNS4_1151	0UNS4	1151	Terracidiphlus	GH30_3(52-466)	GH30_3	Y(1-23)	Xylan
0UNS5_1117	0UNS5	1117	Terracidiphlus	GH30_3(53-467)	GH30_3	Y(1-23)	Xylan
0BWT2_3691	0BWT2	3691	Terracidiphlus	GH30_3(58-468)	GH30_3	Y(1-22)	Xylan
0BWT1_2711	0BWT1	2711	Terracidiphlus	GH30_5(40-475)	CBM13+GH30_	N	Xylan
0UNS5_59	0UNS5	59	Terracidiphlus	GH67(28-702)	GH67	Y(1-27)	xylan
10BWT4_1358	10BWT4	1358	Terracidiphilus	GH67(38-714)	GH67	Y(1-39)	xylan
0BWT2_617	0BWT2	617	Terracidiphlus	GH8(32-367)	GH8	Y(1-27)	xylan
0UNS5_1350	0UNS5	1350	Terracidiphlus	GH8(35-374)	GH8	Y(1-33)	xylan
10BWT4_2330	10BWT4	2330	Terracidiphilus	CE4(239-370)	CE4	Y(1-33)	Xylan, chitin, peptidoglycan
10BWT4_107	10BWT4	107	Terracidiphilus	GH74(91-202)	GH74	N	xyloglucan
0UNS4_1358	0UNS4	1358	Terracidiphlus	GH74(92-206)+CBI	CBM3+GH74	Y(1-36)	xyloglucan
10BWT4_11	10BWT4	11	Terracidiphilus	GH120(304-393)	GH120	Y(1-21)	Xylooligosaccharides
0BWT1_472	0BWT1	472	Terracidiphlus	GH39(38-460)	GH39	Y(1-36)	xylooligosaccharides, xylobiose
10BWT4_1490	10BWT4	1490	Terracidiphilus	GH39(74-512)	GH39	Y(1-24)	xylooligosaccharides, xylobiose
0BWT2_377	0BWT2	377	Terracidiphlus	GH130(12-353)	GH130	N	β-mannosides
0UNS4_1146	0UNS4	1146	Terracidiphlus	GH130(20-318)	GH130	N	β-mannosides
10UNS1_2652	10UNS1	2652	Holophaga	GH130(259-481)	GH130	N	β-mannosides
5UNS4_1350	5UNS4	1350	Holophaga	GH130(259-481)	GH130	N	β-mannosides
0BWT1_2690	0BWT1	2690	Terracidiphlus	GH130(27-327)	GH130	N	β-mannosides
10BWT4_497	10BWT4	497	Terracidiphilus	GH130(30-329)	GH130	N	β-mannosides
0UNS5_143	0UNS5	143	Terracidiphlus	GH130(31-330)	GH130	N	β-mannosides
0BWT2_909	0BWT2	909	Terracidiphlus	GH130(32-330)	GH130	N	β-mannosides
0BWT1_143	0BWT1	143	Terracidiphlus	GH130(47-388)	GH130	N	β-mannosides
0BWT1_2604	0BWT1	2604	Terracidiphlus	GH130(52-364)	GH130	N	β-mannosides
10AWT2_1505	10AWT2	1505	Holophaga	GH130(83-393)	GH130	Y(1-48)	β-mannosides
10BWT3_2154	10BWT3	2154	Holophaga	GH130(83-393)	GH130	Y(1-48)	β-mannosides
10UNS1_1833	10UNS1	1833	Holophaga	GH130(83-393)	GH130	Y(1-48)	β-mannosides
5AWT2_2491	5AWT2	2491	Holophaga	GH130(83-393)	GH130	Y(1-48)	β-mannosides
5BWT7_430	5BWT7	430	Holophaga	GH130(83-393)	GH130	Y(1-48)	β-mannosides
10AWT2_4688	10AWT2	4688	Holophaga	GH130(9-307)	GH130	N	β-mannosides
