# Supplementary material for: Genome-Resolved Metagenomics Informs the Functional Ecology of Uncultured Acidobacteria in Redox Oscillated Sphagnum Peat
Source: mSystems. 2022 Aug 29;7(5):e00055-22. doi: 10.1128/msystems.00055-22 (PMC9599518; doi:10.1128/msystems.00055-22)
Supplement: TABLE S2 [file msystems.00055-22-s0005.pdf]

Table S2: Hydrogenase classifications, neighborhood comparisons, and potential functional roles. All analyses are based on Greening et al., 2016.

Hydrogenase Group	Function	O2 sensitivity	Present in (Genus)	Organization
[FeFe] Group A3	Bifurcating: electrons from H2 to NAD/Ferredoxin; Fermentative H2 evolution	O2 labile	Holophaga	NuoE-Hyd-NuoF
[FeFe] Group A3	Bifurcating: electrons from H2 to NAD/Ferredoxin; Fermentative H2 evolution	O2 labile	Holophaga - only in 5AWT5	HydB-HydA-HydC
[FeFe] Group B	Unconfirmed. May couple fermentative hydrogen evolution to Fd reoxidation.	O2 labile	Holophaga	Catalytic HydM
[FeFe] Group C	Potentially sensory. Phosphatase-linked.	O2 labile	Holophaga - only in 5AWT5	Catalytic HydS--phosphatase--FAD-binding oxidoreductase
[NiFe] Group 1c	H2 uptake. Hydrogenotrophic respiration of fumarate, sulfate, or metals.	O2-sensitive	Terracidiphilus - only in 0UNS4	HybO-HydA-HydB-HydC
[NiFe] Group 1d	H2 uptake. Hydrogenotrophic respiration of O2 or fumarate.	O2-sensitive	Terracidiphilus	HyaC-HyaB-HyaA
[NiFe] Group 3b	Couples fermentative H2 evolution to NADPH oxidation. May be a sulfhydrogenase.	O2-tolerant	Terracidiphilus -- only in 0BWT1	HyhL-HyhS-HyhG-HyhB
[NiFe] Group 3d	Couples H2 oxidation to NAD reduction or in reverse, fermentative NADH-dependent evolution of H2	O2-tolerant	Terracidiphilus	HoxH-HoxY-HoxU-HoxF-HoxE
[NiFe] Group 4b	Respiration involving formate.	O2-sensitive	Terracidiphilus -- only in 0UNS4	HydN-HydM-Formate/nitrite transporter-aldehyde/fd oxidoreductase family -Na(+) H(+) antiporter-HydB-HydG-HydC-HydH-HydK-HydF-HydI-formate dehydrogenase
[NiFe] Group 4d	Respiratory: couples ferredoxin oxidation with proton reduction.	O2-sensitive	Terracidiphilus - 0BWT1, 0BWT2	MbhJ-MbhC-MbhF-MbhM-MbhB
[NiFe] Group 4g	Unconfirmed. May couple respiratory H2 evolution by coupling ferredoxin oxidation to proton reduction.	O2-sensitive	Terracidiphilus - only in 0UNS4	Membrane associated oxidoreducase-hypothetical protein-HydS -hypothetical protein-HydE-HydF-HydG-redox-sensign transcriptional repressor
